# Phosphoproteomic Profiling Reveals Early Salt-Responsive Mechanisms in Two Foxtail Millet Cultivars

**DOI:** 10.3389/fpls.2021.712257

**Published:** 2021-09-20

**Authors:** Jiaowen Pan, Zhen Li, Qingguo Wang, Yanan Guan, Xiaobo Li, Yongguan Huangfu, Fanhua Meng, Jinling Li, Shaojun Dai, Wei Liu

**Affiliations:** ^1^Shandong Academy of Agricultural Sciences, Jinan, China; ^2^College of Life Sciences, Shandong Normal University, Jinan, China; ^3^Key Laboratory of Saline-alkali Vegetation Ecology Restoration (Northeast Forestry University), Ministry of Education, College of Life Sciences, Northeast Forestry University, Harbin, China; ^4^Development Center of Plant Germplasm Resources, College of Life Sciences, Shanghai Normal University, Shanghai, China

**Keywords:** foxtail millet, salt stress, proteomic and phosphoproteomic, phosphorylation, variety innovation and cultivation

## Abstract

Excess soluble salts in saline soils are harmful to most plants. Understanding the biochemical responses to salts in plants and studying the salt tolerance-associated genetic resources in nature will contribute to the improvement of salt tolerance in crops. As an emerging model crop, foxtail millet (*Setaria italica* L.) has been regarded as a novel species for stress resistance investigation. Here, the dynamic proteomic and phosphoproteomic profiling of two foxtail millet varieties of An04 and Yugu2 with contrasting salt tolerance characteristics were investigated under salt stress. In total, 10,366 sites representing to 2,862 proteins were detected and quantified. There were 759 and 990 sites corresponding to 484 and 633 proteins identified under salinity in An04 and Yugu2, respectively, and 1,264 and 1,131 phosphorylation sites corresponding to 789 and 731 proteins were identified between these two varieties before and after salt stress, respectively. The differentially-regulated phosphoproteins (DRPPs) were mainly involved in signal transduction, regulation of gene expression, translation, ion transport, and metabolism processes. Yugu2 possessed signal perception and transduction capabilities more rapidly and had a more intense response compared with An04 upon salinity. The sucrose metabolism pathway, in particularly, might play a vital role in salt response in foxtail millet, which not only provides UDP-glucose for the cellulose synthesis and energy production, but also promotes flavonoid related synthesis to enhance the salt tolerance ability. Over-expressing the phospho-mimic sucrose synthase (SuS) (*SuS*^*S*10*D*^) in soybean roots enhanced salt tolerance compared with over-expressing *SuS* lines. The knowledge of this research will shed light on elucidating the mechanisms of salt response, and pave the way for crop varieties innovation and cultivation under salinity and stresses.

## Introduction

Protein reversible phosphorylation is the most widespread protein post-translational modification (PTM) and it affects nearly all cellular processes including signal perception and transduction, transcription, translation, as well as metabolic reactions (Hsu et al., 2018). In general, protein phosphorylation and dephosphorylation occur mainly at serine, threonine, and tyrosine residues and are catalyzed by kinases and phosphatases. The phosphorylation modification on specific sites of a protein can regulate protein configuration and ultimately modify their functions and enzyme activity, structure stability, substrate specificity, and intracellular localization, even without changing the protein and mRNA transcript level (Lv et al., 2016). Isobaric tags for relative and absolute quantitation-based (iTRAQ) and tandem mass tags-based (TMTs) quantitative proteomics approaches are widely used for quantitative analysis of protein phosphorylation. Large numbers of proteomic phosphorylation analyses have been performed in different plant species such as bermudagrass (*Cynodon dactylon*) stolons, tomato (*Lycopersicon esculentum*), okra (*Abelmoschus esculentus* (*linn.*) *Moench*), sugar beet (*Beta vulgaris*), maize (*Zea mays*), and wheat (*Triticum aestivum*), in response to various abiotic stresses, and large number of stress-induced phosphoproteins were identified (Hu et al., 2015; Lv et al., 2016; Yu et al., 2016, 2019; Hsu et al., 2018; Zhang et al., 2019). In sugar beet monosomic addition line M14, phosphoproteomics analysis found that the protein phosphorylation events are not only involved in signal transduction, but also spread across key physiological processes such as stress and defense, transcription, transport, and metabolism (Yu et al., 2016). In soybean, quantitative phosphoproteomics study revealed that the salt activated phosphorylation of GmMYB173 increases the expression of *GmCHS5*, which contributes to the accumulation of flavonoids and enhances salt tolerance of plants (Pi et al., 2018).

Soil salinity is a global problem that affect crops productivity and threatens food security worldwide. More than one-third of irrigated lands in the world are affected by salinization, and the situation is becoming worse (Zhao et al., 2020). Salinity stress limits plant growth and development by imposing several major restrictions, such as osmotic stress, ionic imbalance, and secondary stress. Osmotic stress caused by excess soluble salts, could reduce the water potential, ultimately resulting in water deficit in plants. Ionic imbalance derived from an uneven accumulation of cations and anions such as Na^+^, K^+^, and Cl^–^ in intracellular compartments, can disturb the uptake and metabolism of other essential ions (Yang and Guo, 2018; Zhao et al., 2020). Accumulation of toxic compounds and nutrient imbalances induced by salt stress were termed as secondary stresses (Yang and Guo, 2018).

Plants have evolved the ability to sense salt level, but the Na^+^ sensor or receptor is not fully understood. Osmotic and ionic stresses trigger elevated cytosolic Ca^2+^ concentration within seconds to minute after exposure. The channel proteins of Mono cation-induced [Ca^2+^]_*i*_ increases 1 (MOCA1), plastid K^+^ exchange antiporters (KEAs), and annexin1 (ANN1) have also been reported to mediate salt-induced Ca^2+^ signals (Zhao et al., 2020). In addition to Ca^2+^ signals, the salt stress has also been reported to be sensed by the cell wall. Three leucine-rich repeat receptor kinases (LRR-RKs) of *Arabidopsis* act as key components of cell wall integrity by being able to sense and respond to salt stress (Xu et al., 2008; Van der Does et al., 2017). A recent study has indicated that the cell wall-localized leucine-rich repeat extensions (LRXs), together with secretory peptides RALFs and the receptor-like kinase FER, could function as a module to sense cell-wall signals and regulate salt stress tolerance (Zhao et al., 2018). A Ca^2+^ signal triggered by salt stress was decoded by diverse calcium-dependent proteins, such as calcium-dependent protein kinases (CDPKs), calcineurin B-like proteins (CBLs) and CBL-interacting protein kinases (CIPKs). Following activation of the signal, the salt stress signal transduction and regulatory pathways such as SOS signaling pathways, MEKK1-MKK2-MPK4/MPK6 and MKK9-MPK3/MPK6 cascades, and ABA-dependent signaling pathways are activated (Julkowska and Testerink, 2015). Plants possess two major methods to maintain cellular ion homeostasis. One is reducing contents of cytoplasmic Na^+^ by restricting Na^+^ uptake and promoting Na^+^ efflux via activation of various ion channels. Another is vacuolar Na^+^ sequestration. Compatible osmolytes, such as proline, glycine betaine, sugars, and polyamines, could also be synthesized and accumulated to reduce the osmotic potential of plants under salinity (Yang and Guo, 2018).

Most crop species are glycophyte and are not able to complete their life cycle upon excess salt concentrations (Yang and Guo, 2018). Thus, understanding how salinity affects the biochemical, physiological, metabolic, and morphological properties of plants is of paramount importance for improving salinity stress tolerance of crops. Foxtail millet (*Setaria italica* L.) is a drought tolerance and nutritious cereal that owns strategic positions in the national economy and social development in China, India, and Africa. The potential of *Setaria* as a model system is primarily based on its attributes for genetic analysis, particularly the small diploid genome, small physical stature, C_4_ photosynthetic capability, transformability, and a growing list of genetic and genomic resources (Zhang et al., 2012; Lata et al., 2013). With the world dwindling water resources and increasing soil salinity, foxtail millet as an emerging model crop and strategic reserve grain is often exposed to salinity and stress (Lata et al., 2013). Improving salt tolerance is one of the main objectives for foxtail millet cultivation and breeding.

Recently reports have shown that Yugu2 was salt tolerant variety and An04 was salt sensitive variety (Pan et al., 2020). Our previous integrative transcriptomics and metabolomics analyses indicated that several key biological processes, such as ion transport, redox homeostasis, and specific biosynthetic pathways including of flavonoids and lysophospholipids were crucial for salt tolerance in foxtail millet (Pan et al., 2020). However, the role of global protein phosphorylation modifications and its dynamics in regulating response of foxtail millet to salt stress seldom researched.

In this study, comprehensive proteomic and phosphoproteomic analysis was performed to explore sophisticated responsive networks of foxtail millet to salinity stress. A large number of phosphorylation sites and phosphoproteins involved in the salt stress response were identified. Our results showed that phosphoproteins involved in signal perception and transduction, chromatin remodeling, transcription and translation processes and sucrose metabolism might play vital roles in salt stress signaling and response in foxtail millet. Particularly, the sucrose metabolism pathway might play a central role in the salt response of foxtail millet, which not only provides UDP-glucose for the cellulose synthesis and energy production, but also promotes flavonoid synthesis to enhance the salt tolerance. This work is a first step elucidating the early signaling and sodium-sensing machinery of foxtail millet, and will provide new perspectives for quality improvement and breeding of crops under salinity.

## Materials and Methods

### Plant Materials, Growth Conditions and Physiological Measurements

Foxtail millet (*Setaria italica* L.) seedlings were cultivated hydroponically as described before with modifications. The salt tolerance variety Yugu2 and salt sensitive variety An04 were used in this study (Pan et al., 2020). Briefly, sterilized seeds of Yugu2 and An04 were germinated on moist filter paper in petri dishes for 12 h. Seedlings were transferred to hydroponic containers containing the 1/2 Hoagland solution placed in a light chamber under 30/25°C day/night cycle, 450 μmol m^–2^ s^–1^ light intensity, and relative humidity of 70% with a 14 h photoperiod. After two weeks, the seedlings were treated with 150 mM NaCl solutions. The roots were sampled for physiological measurements at 0, 15, 30, 45, and 60 min after been exposed to salt with three biological replicates. The activities of antioxidant enzymes were measured as described previously (Pan et al., 2020). For proteomic and phosphoproteomic analysis, the two weeks old seedlings were treated with 150 mM NaCl for 0 min and 15 min, and the roots were collected and washed gently with sterile water, then frozen in liquid nitrogen for further analysis. Three independent biological replications were performed for each sample.

### Protein Extraction and Trypsin Digestion

The roots of foxtail millet were homogenized in liquid nitrogen. Then four volumes of lysis buffer (8 M urea, 10 mM dithiothreitol, 1% Triton-100, and 1% Protease Inhibitor Cocktail) was added to each sample. Samples were then sonicated for three times on ice using an ultrasonic processor (Scientz). After centrifugation with 20,000 g at 4°C for 10 min, the debris was removed. The protein was precipitated with cold 20% TCA and washed with cold acetone three times. The protein was redissolved in 8 M urea and the total protein concentration was measured using a BCA kit according to the manufacturer’s protocol. For trypsin digestion, the protein solution was reduced with 5 mM dithiothreitol at 56°C for 30 min and alkylated with 11 mM iodoacetamide at room temperature in darkness for 15 min, then diluted with 100 mM TEAB. The trypsin was added at 1:50 trypsin-to-protein mass ratio for the first digestion overnight and 1:100 trypsin-to-protein mass ratio for a second 4 h-digestion.

### TMT Labeling and HPLC Fractionation

After trypsin digestion, the resulting peptide mixture was desalted by Strata X C18 SPE column (Phenomenex) and vacuum-dried. Peptide mixtures were reconstituted in 0.5 M TEAB and processed according to the manufacturer’s protocol for TMT kit. Briefly, one unit of TMT/iTRAQ reagent were thawed and reconstituted in acetonitrile. The peptide mixtures were then incubated for 2 h at room temperature and pooled, desalted and dried by vacuum centrifugation.

The samples were fractionated according to the protocol (Pan et al., 2018). The tryptic peptides were fractionated into fractions by high pH reverse-phase HPLC using Thermo Betasil C18 column (5 μm particles, 10 mm ID, 250 mm length). Briefly, peptides were first separated with a gradient of 8% to 32% acetonitrile (pH 9.0) over 60 min into 60 fractions. Then, the peptides were combined into nine fractions for proteomic analysis and six for phosphoproteomic analysis, and then dried by vacuum centrifuging.

### Affinity Enrichment for Phosphoproteomic Analysis

Peptide mixtures were first incubated with IMAC microspheres (Thermo, A32992) suspension with vibration in loading buffer (50% acetonitrile/0.5% acetic acid). The IMAC microspheres with enriched phosphopeptides were then collected by centrifugation, and the supernatant was removed. To remove non-specifically adsorbed peptides, the IMAC microspheres were washed with 50% acetonitrile/0.5% acetic acid and 30% acetonitrile/0.1% trifluoroacetic acid, sequentially. To elute the enriched phosphopeptides from the IMAC microspheres, elution buffer containing 10% NH_4_OH was added and the enriched phosphopeptides were eluted with vibration. The supernatant containing phosphopeptides was collected and lyophilized for LC-MS/MS analysis.

### LC-MS/MS Analysis

LC-MS/MS analysis was carried out as described previously (Pan et al., 2018). The tryptic peptides were dissolved in 0.1% formic acid (solvent A), then directly loaded onto a home-made reversed-phase analytical column (15-cm length, 75 μm i.d.). The samples were resolved on UPLC with xyz columncoupled on line with a Q Exactive^TM^ Plus hybrid quadrupole-orbitrap mass spectrometer equipped with NSI source. The electrospray voltage adopted was 2.0 kV. The m/z scan range was 350 to 1800 for full scan, and intact peptides were identified in the Orbitrap at a resolution of 70,000. Peptides were then selected for MS/MS using NCE setting as 28 and the fragments were detected in the Orbitrap at a resolution of 17,500. A data-dependent procedure that alternated between one MS scan followed by 20 MS/MS scans with 15.0s dynamic exclusion. Automatic gain control (AGC) was set at 5E4. Fixed first mass as 100 m/z.

### Database Search

The resulting MS/MS data was processed as previously described (Pan et al., 2018). Briefly, the data were processed using Mascot search engine (v.2.3.0). Tandem mass spectra were blasted against *Uniprot_foxtail_4555*^1^ database concatenated with reverse decoy database. Trypsin/P was specified as the cleavage enzyme allowing up to four missing cleavages. The mass tolerance for precursor ions was set as 20 ppm in First search and 5 ppm in Main search, and the mass tolerance for fragment ions that set as 0.02 Da. FDR was adjusted to <1%, and the minimum score for modified peptides was set to >40. For phosphorylation site analysis, the lowest available underscore intensity entries from the MaxQuant output were used. For phosphorylation localization, the lowest MaxQuant-calculated localization probability per method was used to filter confidently localized phosphorylation sites with a threshold of >0.75 (Hogrebe et al., 2018). Data are available via Proteome Xchange with identifier PXD025969. MaxQuant output file Phospho Sites (such as S, T, Y) txt was used to analysis of phosphorylation sites quantification. In this study, the quantitative values of each sample in three replicates were obtained by three full quantitative experiments. The first step is to calculate the differentially modified phosphosites between the two samples. Firstly, calculate the average value of the quantitative values of each sample in three replicates, and then calculate the ratio of the average values between the two samples. The ratio is used as the final quantitation. The second step is to calculate the significant *p*-value of differential expression between two samples. Firstly, the relative quantitative values of each sample were taken as log2 transform (so that the data conforms to the normal distribution), and *p* value was calculated by the two-sample two-tailed *T*-test method. *P*-value < 0.05 and protein ratio >1.2 was regarded as up regulation, and *p*-value < 0.05 and protein ratio < 0.833 was regarded as down-regulation. Raw abundance ratios of phosphorylation sites were normalized based on the corresponding proteins ratio.

### Bioinformatics Analysis

The identified and differentially expressed protein and phosphoprotein sequences were mapped with Gene Ontology Terms^2^. The identified proteins and phosphoproteins were classified based on the biological process, molecular function and cellular component. The COG database^3^ was employed for the clusters of orthologous proteins. The Kyoto Encyclopedia of Genes and Genomes (KEGG) database^4^ was adopted for pathway analysis. The pathway enrichment statistics were performed by Fisher’s exact test, and those with a corrected *p* < 0.05 were considered the most significant pathways. Wolf-psort^5^ was used to predict the protein’s subcellular localization. The protein-protein interaction network was analyzed using the online STRING 10.5 tool^6^ and visualized by Cytoscape (3.2.0). The enrichment-based clustering was based on different protein functional classification according to Bonhomme et al. (2012). Phosphorylation motifs of the differentially regulated phosphoproteins (DRPPs) were analyzed using motif-X software according to Lu et al. (2019). The K-means method was adopted to analyze and cluster the identified proteins and phosphoproteins according to Pan et al. (2020).

### Rapid Function Test of Sucrose Synthase (SuS) in Soybean Hairy Root System

The full-length coding sequence of foxtail millet *SUS* gene (accession numbers: K3XVC3) was amplified using primer sets forward TGAGCCATGGCTTCCAAGCTGA and reverse CGATGGTTCTCCCGCTTGATG from cDNA. The resulting fragment was cloned in pEASY-Blunt3 Cloning vector (Transgen biotech, Beijing) and sequenced. The phospho-mimic (*SuS*^*S*10*D*^) mutant was generated using overlap extension PCR as previously described (Pan et al., 2012). The full length of verified coding regions of *SuS* and *SuS*^*S*10*D*^ were separately ligated into the Pex33-DsRed-MCS-DHA vector, which included RFP tag and DHA tags between *Spe*I and *Kpn*I sites. The empty Pex33-DsRed-MCS-DHA vector was used as a negative control. The empty vector and the *SuS* and *SuS*^*S*10*D*^ constructs were then transformed into the cotyledonary soybean cultivar of node of Qihuang 34 via *Agrobacterium rhizogenes* strain K599 infection (Pi et al., 2018). After two weeks of co-cultured, the transgenic roots could be detected using fluorescent stereo microscope (OLYMPUS). The screened positive transgenic roots with the same length were transferred to the MS medium with or without 150 mmol NaCl and allowed to grow for 5 additional days. The H_2_O_2_ in the roots were qualitatively estimated using DAB (3,3’-diaminobenzidine) assay as previously described (Pan et al., 2020).

### Statistical Analysis

Statistical analysis was carried out using SPSS13.0 software. All the results were presented as means ± standard deviation of at least three replicates.

## Results

### The Activities of Antioxidant Enzymes in Two Foxtail Millet With Contrasting Salt Tolerance Under Salt Stress

**FIGURE 1:**
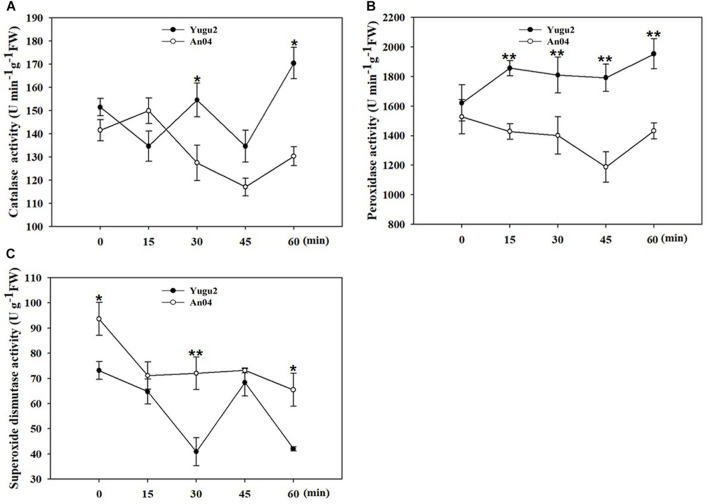
Measurement of antioxidant enzymes activities of catalase (CAT) **(A)**, peroxidase (POD) **(B)**, and superoxide dismutase (SOD) **(C)**. Error bars indicate ± SE of at least three biological repeats. Student’s *t*-test were carried out to analyze significant differences between two varieties, **indicates the *p*-value < 0.01 and *indicates the *p*-value < 0.05, respectively.

Our previous research identified foxtail millet cultivar, An04 as more salt sensitive and Yugu2 as more salt tolerance (Pan et al., 2020). To determine the appropriate sampling time for phosphoproteomic analysis at the onset of response to salt stress, the activities of antioxidant enzymes were measured over time after the seedlings were exposed to 150 mM NaCl solution. As shown in Figure 1, the activities of catalase (CAT), peroxidase (POD) and superoxide dismutase (SOD) displayed significant differences between Yugu2 and An04. The overall activities of CAT and POD in Yugu2 were higher than those of An04 within 60 min under 150 mM NaCl, except an opposite trend of CAT activity occurred at 15 min under salinity (Figures 1A,B). The overall SOD activity of An04 was higher than that of Yugu2, while it showed slightly decreased under salinity (Figure 1C). Referring to the data above, the 15 min time point of 150 mM NaCl treatment was assessed as the optimum sampling time for proteomic and phosphoproteomics analysis.

### Identification of Differential Abundant Proteins and Phosphoproteins in Yugu2 and An04 Under Salinity

The root samples of An04 and Yugu2 were named AN04C and YG2C for untreated control and AN04T and YG2T for salt treatment, respectively. A total of 39,053 phosphorylation sites of 6,278 proteins were identified in the roots of Yugu2 and An04. To evaluate the quality of the biological replicates of each sample, a principal component analysis (PCA) was performed. The results revealed that the biological replicates of each sample clustered together in separated areas, indicating that the two foxtail millet varieties have distinct phosphoproteomic profiles (36.1%, PC1), and salt stress had rewired the signal transduction of the two varieties (22.7%, PC2) (Supplementary Figure 1). To ensure that the data was highly reliable, a criterion of a localization probability of >0.75 was adopted to filter the data, resulting in the detection and quantification of 17,553 unique phosphorylation sites mapping to 5,595 phosphoproteins. The identified sites and proteins were also normalized by the proteome, and ultimately 10,366 sites of 2,862 proteins provided quantitative information (Table 1 and Supplementary Table 1).

**TABLE 1:** Basic statistical table of MS results.

Total spectrum	Matched spectrum	Peptides	Modified peptides	Identified proteins	Identified sites	Quantifiable proteins	Quantifiable sites	Normalized proteins	Normalized sites
599551	111811 (18.6%)	45242	39053	6278	25230	5595	17553	2862	10366

*The proteins and modification sites filtered with localization probability > 0.75. Identified proteins: Number of proteins detected by spectrum search analysis, Identified sites: Number of modification sites detected by spectrum search analysis, Quantifiable proteins: Number of proteins quantifiable, Quantifiable sites: Number of modification sites quantifiable, Normalized proteins: Number of modification proteins quantifiable after normalized by proteome, Normalized sites: Number of modification sites quantifiable after normalized by proteome.*

**FIGURE 2:**
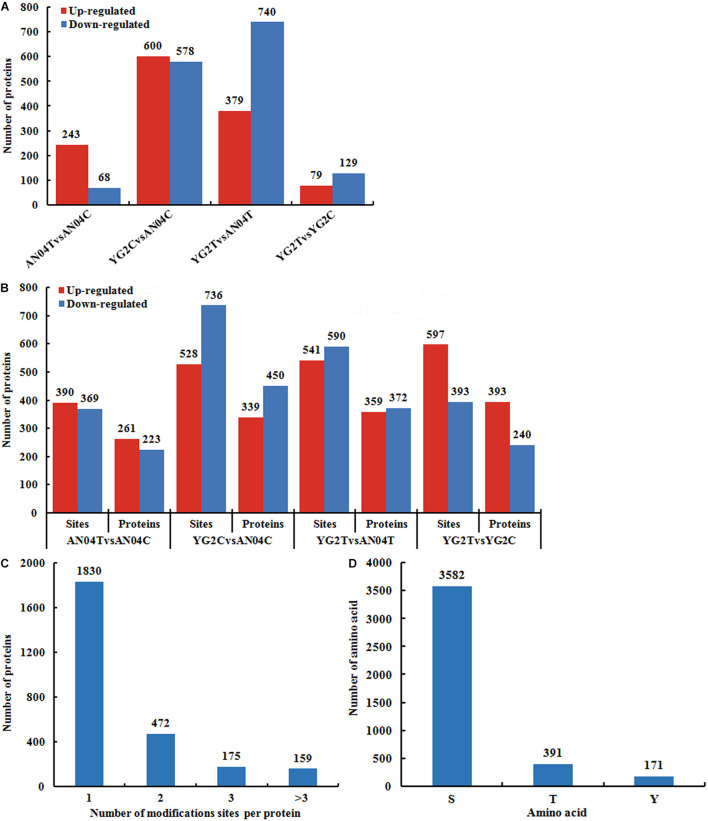
Overview of the identified proteins and phosphorylation sites in An04 and Yugu2 in response to salt stress. The numbers of DAPs **(A)**, DRPPs and phosphorylation sites **(B)** in different comparison groups. **(C)** Distribution of proteins containing different numbers of phosphorylated sites. **(D)** Distribution of phosphorylated amino acids.

Filtered with the threshold value of expression fold change of >1.2 in protein abundance and the *P* < 0.05, 311 differentially abundant proteins (DAPs) were identified in An04 and 208 in Yugu2 in response to salt stress. There were 789 DAPs and 731 DAPs identified between two varieties before and after salt treatment (YG2CvsAN04C and YG2TvsAN04T), respectively (Figure 2A and Supplementary Table 2). The phosphorylation level of the site in the differentially regulated phosphoproteins (DRPPs)was filtered by the threshold value of 1.2-fold, a ratio of > 1.20 and < 0.83 (*P*-value < 0.05) were considered as increased and decreased DRPPs, respectively.

In An04, there were 759 (390 experienced an increase and 369 experienced a decrease in phosphorylation levels) sites corresponding to 484 (261 increased and 233 decreased) proteins identified after salt stress. There were 990 (597 experienced an increase and 393 experienced a decrease in phosphorylation levels) sites corresponding to 633 (393 increased and 240 decreased) proteins identified in Yugu2 under salinity (Figure 2B and Supplementary Table 3). Between these two varieties, there were 1264 (528 experienced an increase and 736 experienced a decrease in phosphorylation levels) phosphorylation sites corresponding to 789 (339 increased and 450 decreased) proteins identified before salt stress (YG2CvsAN04C). There were 1131 (541 experienced an increase and 590 experienced a decrease in phosphorylation levels) phosphorylation sites corresponding to 731 (359 increased and 372 decreased) proteins identified after salt stress (YG2TvsAN04T) (Figure 2B and Supplementary Table 3). Notably, most phosphoproteins contained only one phosphorylation site, while 472, 175, and 159 phosphoproteins contained two, three or multiple phosphorylation sites, respectively (Figure 2C). Among the phosphorylation sites, most of them (3582 amino acids) were phosphorylated at serine residues, while 391 at threonine residues, and 171 at tyrosine residues (Figure 2D).

### KOG Annotation and Classification of the DAPs and DRPPs

To carry out functional analysis, all the DAPs and DRPPs were mapped to 24 categories in the Clusters of Orthologous Groups of proteins (KOG/COG) database. Analysis of the identified DAPs suggested that posttranslational modification, protein turnover, chaperones (O), secondary metabolite biosynthesis, transport (Q), translation, ribosomal structure and biogenesis (J), carbohydrate transport and metabolism (G), and energy production and conversion (C) were the top 5 functional categories (Supplementary Figure 2A and Supplementary Table 4). The identified DAPs were mainly localized in the chloroplasts, cytoplasm, extracellular, nucleus, and plasma membrane (Supplementary Figure 2B and Supplementary Table 4).

**FIGURE 3:**
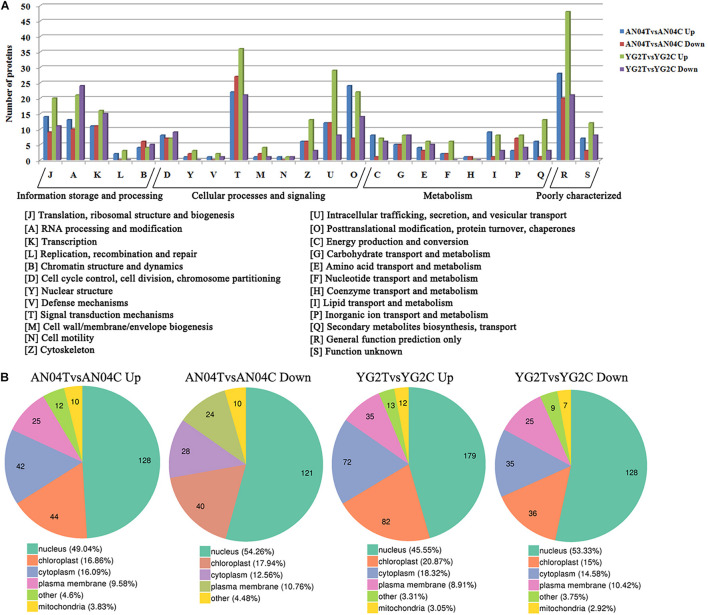
**(A)** The clusters of orthologous groups of proteins (KOG/COG) classification of the DRPPs in Yugu2 and An04 after salt stress. **(B)** The subcellular localization of DRPPs in Yugu2 and An04 after salt stress.

KOG analysis for the DRPPs indicated that signal transduction mechanisms (T), Intracellular trafficking, secretion, and vesicular transport (U), posttranslational modification, protein turnover, chaperones (O), translation, ribosomal structure and biogenesis (J), RNA processing and modification (A), and transcription (K) were overrepresented functional categories (Figure 3A, Supplementary Figure 3A and Supplementary Table 5). Over 40% of DRPPs were predicted to be associated with the nucleus, and the remainder mainly localized in chloroplast, cytoplasm and plasma membrane (Figure 3B, Supplementary Figure 3B and Supplementary Table 5).

### Enrichment-Based Clustering of DAPs and DRPPs

To further characterize the functions of DAPs and DRPPs, the hierarchical clustering based on different protein functional classification (such as the GO and KEGG Pathway) was performed, and the results indicated the change of the co-expression trends of DAPs and DRPPs between the groups.

Comparison of the DAPs between the two salt-tolerant and salt-sensitive varieties (YG2CvsAN04C) showed that the DAPs were mainly focused on the processes of intracellular protein transport, regulation of signal transduction, cellular carbohydrate biosynthesis, polysaccharide biosynthesis, secretion, and regulation of protein catabolism (Supplementary Figure 4 and Supplementary Table 6).

Comparison of AN04T with AN04C showed that the increased DAPs in response to salt stress in this salt-sensitive cultivar were enriched in the following processes: pentose metabolism, photosynthesis, monosaccharide and polysaccharide metabolism, defense response, and lipid transport (Supplementary Figure 4 and Supplementary Table 6). On the other hand, the increased DAPs of the salt-tolerant Yugu2 cultivar in response to salt stress (YG2CvsYG2T) were mainly enriched in the following processes: organic acid biosynthesis, carboxylic acid metabolism, and oxoacid metabolism, while the proteins in amine biosynthetic and organic acid catabolic processes were decreased dramatically. In view of the outstanding salt tolerance of Yugu2, the results above indicated that accumulation of organic acid may contribute to the enhancement of the salt tolerance in foxtail millet under salinity (Supplementary Figure 4 and Supplementary Table 6).

In response to salt stress, the salt-tolerant and salt-sensitive varieties showed differential DAPs. The increased DAPs in YG2TvsAN04T were mainly enriched in lignin catabolic process, chromosome organization, cellular macromolecular complex assembly, and DNA packaging (Supplementary Figure 4 and Supplementary Table 6). The S-adenosylmethionine (SAM) biosynthetic process was enriched in the increased DAPs in the salt-tolerant cultivar (Yugu2) without (YG2CvsAN04C) or with salt stress (YG2TvsAN04T) (Supplementary Figure 4 and Supplementary Table 6). SAM has been widely studied for its role in controlling plant development and stress response, it provides methyl groups for the methylation of DNA, RNA, proteins, and lipids and is a common precursor of polyamines (PAs) and ethylene, both of which play critical roles in regulating stress response in plants (He et al., 2019).

Differentially regulated phosphoproteins analysis showed drastic differences in pattern of protein phosphorylation between the two varieties without or with salt stress. Without salt stress, the up-regulated DRPPs in salt-tolerance YG2C vs salt-sensitive AN04C were mainly clustered in the processes of glucan biosynthesis, polysaccharide biosynthesis, carbohydrate biosynthesis, monovalent inorganic cation transport and peptide biosynthesis. The protein complex disassembly was mainly concentrated in decreased DRPPs.

**FIGURE 4:**
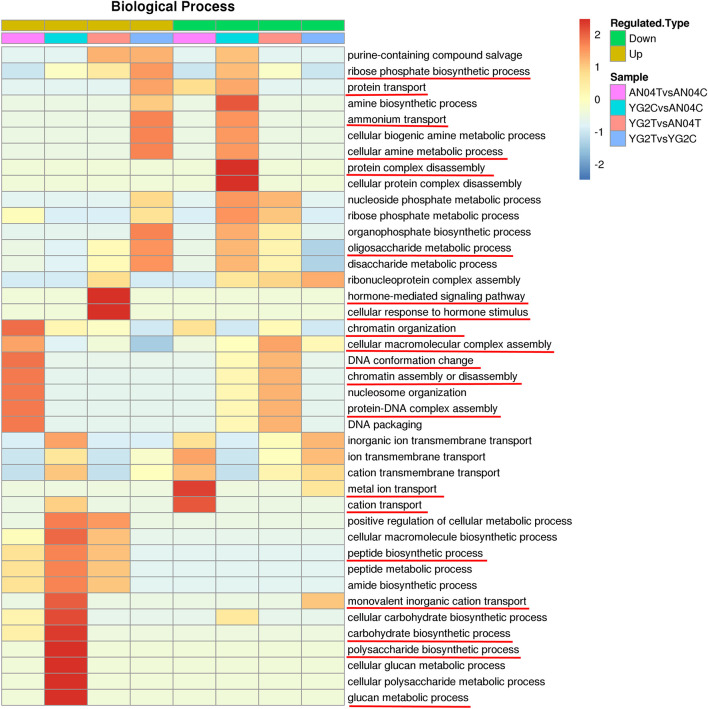
GO functional cluster of DRPPs in the biological process. The key biological processes were marked with red lines.

Salt stress in the salt sensitive cultivar (AN04TvsAN04C) resulted in enrichment of increased DRPPs related to the chromatin organization, cellular macromolecular complex assembly, DNA conformation change, chromatin assembly or disassembly, and protein-DNA complex assembly (Figure 4 and Supplementary Table 7), while, these biological process categories were enriched in the decreased DRPPs when the salt-tolerance cultivar was compared with the salt-sensitive cultivar under salinity, the YG2TvsAN04T comparison (Figure 4 and Supplementary Table 7).

The DRPPs related to metal ion transport and cation transport were distinctively enriched in the decreased DRPPs of An04 (Figure 4 and Supplementary Table 7), which may be the reason for the salt sensitivity of An04. In salt-tolerant cultivar, Yugu2, salt stress increased the DRPPs of proteins that participated in ribose phosphate biosynthetic process, protein transport, cellular amine metabolic process, ammonium transport, and oligosaccharide metabolic process.

The proteins involved in response to hormone-mediated signaling pathway and cellular response to hormone stimulus were enriched in the up-regulated DRPPs of YG2TvsAN04T, which indicate that the phytohormones may play a vital role in response to salt stress.

### K-Means Clustering, Functional Enrichment and Motif Analysis of Proteins and Phosphoproteins

**FIGURE 5:**
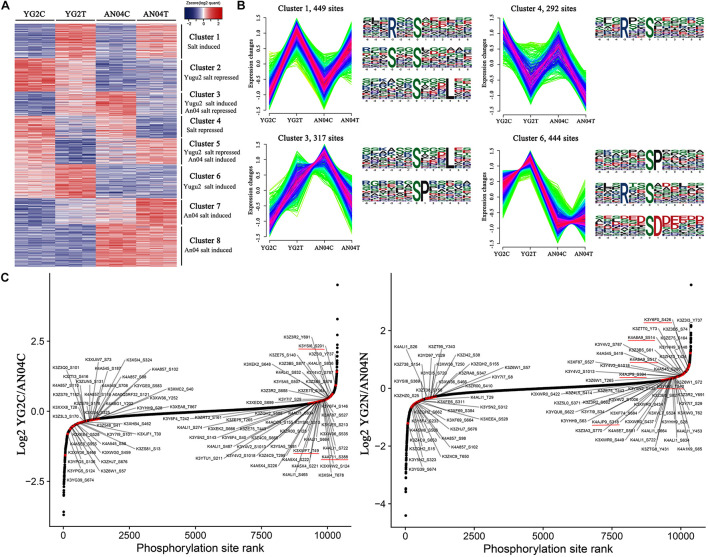
**(A)** Hierarchical clustering of DRPPs under salt stress of foxtail millet. The color code of each phosphorylation site (row) in all samples (columns) indicates the low (blue) and high (red) Z-score normalized intensities. **(B)** K-means clustering and phosphorylation motifs analysis of phosphorylation sites in Yugu2 and An04 during salt stress. **(C)** Salt stress regulates the phosphorylation sites on foxtail millet kinases. Ranked list of phosphorylation changes of kinases in response to salt treatment. The key kinases were marked with red lines.

To investigate the variation trends of proteins and phosphoproteins of AN04C, AN04T, YG2C, and YG2T, a k-means clustering analysis was performed. Eight clusters of proteins and phosphoproteins that exhibited unique expression profiles were subjected to further analysis (Figure 5, Supplementary Figures 5, 6). The proteins of cluster 1 and cluster 5 with more abundance in the samples of An04 (Supplementary Figure 5 and Supplementary Table 8) were mainly involved in carbohydrate transport and metabolism, translation, ribosomal structure and biogenesis and posttranslational modification, protein turnover, and protein folding (chaperones). Cluster 2 contained 183 proteins and cluster 8 contained 293 proteins, exhibiting preferential expressions in the salt-tolerant Yugu2 cultivar. These proteins participated in posttranslational modification, protein turnover, chaperones, signal transduction mechanisms, intracellular trafficking, secretion, and vesicular transport and secondary metabolites biosynthesis, transport, and catabolism (Supplementary Figure 5 and Supplementary Table 8). These results corroborate with our previous RNA-Seq results, and suggest the highlight processes may be critical for the salt tolerance in foxtail millet (Pan et al., 2020).

For phosphoproteins, the phosphorylation levels of 449 sites in cluster 1 were increased, and 292 sites in cluster 4 were decreased in both cultivars after salt stress (Figures 5A,B, and Supplementary Table 9). These phosphoproteins mainly enriched in RNA processing and modification, energy production and conversion, transcription, post-translational modification, protein turnover, chaperones, inorganic ion transport and metabolism, signal transduction mechanisms, and cytoskeleton, indicating that these processes may participated in stress response regulation in foxtail millet under salinity. The phosphorylation motif analysis shows that the [RxxS], [SxxS], and [SxxxL] motifs are dominant in these clusters (Supplementary Table 10). Further analysis showed that the [RxxS] motif was a potential substrate for CaMK-II (Calmodulin-dependent protein kinase), MAPKK (Mitogen-activated protein kinase kinase), and protein kinase A (Zhong et al., 2017; Liu et al., 2019). Considering the signal transduction and response roles under stress of the proteins mentioned above, the [RxxS] motif supposed to be essential for the salt signaling and response of foxtail millet.

Cluster 3 contained 317 phosphorylation sites that were phosphorylated in Yugu2 but dephosphorylated in An04 (Figures 5A,B, and Supplementary Table 9). These may contribute to the differential salt stress tolerance abilities in these cultivars. These proteins were mainly related to the intracellular trafficking, secretion, vesicular transport, signal transduction, inorganic ion transport and metabolism and sucrose metabolism. The motifs of [SxxxL] and [SP] were enriched in this cluster (Supplementary Table 10). There were 444 sites of proteins in cluster 6 that were specifically phosphorylated in Yugu2 under salinity. These proteins mainly enriched the signal transduction mechanisms, regulation of gene expression, translation, ribosomal structure and biogenesis, PTM, protein turnover, chaperones, and ion transport (Figures 5A,B, and Supplementary Table 9). In addition, a series of protein kinases and transcription factors were also enriched in this cluster (Supplementary Table 10). The motifs of [SP], [RxxS], and [SD] were over-represented in this cluster. As known, most proteins that contain [SP] and [RxxS] motifs are potentially recognized by SnRK1, SnRK2, CDPKs, MAPKs, cyclin-dependent kinase (CDK), and receptor-like kinases (RLKs) (Nikonorova et al., 2018; Lu et al., 2019). In addition, the [SD] motif is an acidic motif, which normally is recognized by casein kinase-II (CK-II) (Zhang et al., 2014; Pi et al., 2018). The enriched phosphorylation motifs and candidate target proteins in clusters 3 and 6 might indicate the quicker and higher efficiency of signal transduction, gene expression regulation, and protein modification abilities in Yugu2 compared to An04.

Considering that the perturbation of kinase activity plays an important role in mediating stress signaling and that phosphorylation of kinases determines the kinase activity, the phosphorylation changes of the phosphorylation sites in identified kinases in two foxtail millet cultivars were compared. The results show that the two cultivars possess unique kinase phosphorylation expression patterns before and after salt stress (Figure 5C and Supplementary Table 11). In Yugu2, the phosphorylation sites in casein kinase (K3YSI6) and CDPK (K4A711 and K3XWF7) presented higher phosphorylation levels before treatment. These kinases might be responsible for the phosphorylation of enriched motifs in cluster 2 (Supplementary Table 11). Upon salt treatment, the 35 phosphorylation sites in 24 kinases were activated in Yugu2 (Supplementary Table 11). These include Thr220 and Tyr222 in the activation loops of Mitogen-activated protein kinase (MPK, K3XXF6) and the Thr191 and Tyr193 of MPK (K3XXQ0), which are homologs of MPK6 (Thr221 and Yyr223) and MPK4 (Thr201 and Tyr203) in *Arabidopsis*, respectively. It is reasonable to assume that there are some similarities in salt stress signaling and tolerance mechanisms between foxtail millet and *Arabidopsis*, in which salt stress activates the MKK1-MKK2-MPK4/MPK6 cascades to mediate plant stress tolerance (Julkowska and Testerink, 2015). In addition, 7 RLKs, 2 CDPKs and 1 CDK were also activated in salt treated Yugu2, which could explain why the [SP], [RxxS], and [SD] motifs were more enriched in the salt-induced phosphorylation sites of cluster 1, 3 and 6 (Figure 5B and Supplementary Table 11).

### Protein-Protein Interaction (PPI) Analysis of DRPPs

**FIGURE 6:**
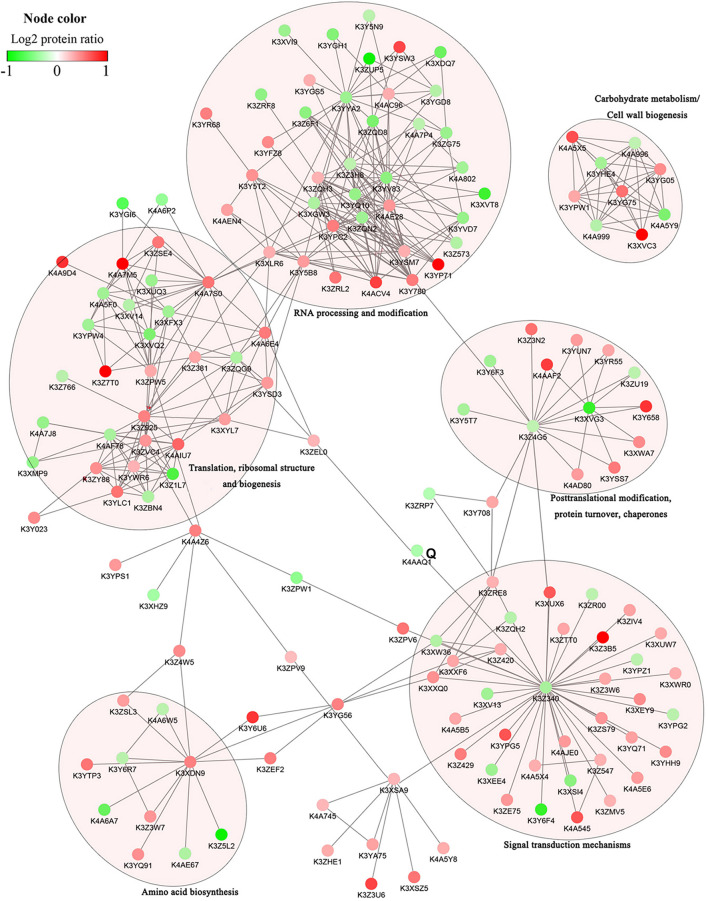
Protein–protein interaction networks of DRPPs in six functional categories of Yugu2.

To investigate how signaling pathways participate in stress signal transduction and the response to salinity in foxtail millet, the DRPPs identified above were further analyzed using the STRING database and Cytoscape software. The PPI networks were constructed, resulting in assembly of 158 nodes in Yugu2 and 82 nodes in An04 (Figure 6, Supplementary Figure 7, and Supplementary Table 12). In these interaction networks, six subnets of significant enrichment in Yugu2, and seven in An04 were identified. Simultaneously, four subnets related to translation, ribosomal structure and biogenesis, RNA processing and modification, post-translational modification, and carbohydrate metabolism/cell wall biogenesis were significantly enriched in both Yugu2 and AN04. In Yugu2, the subnets related to amino acid biosynthesis and signal transduction mechanisms were also enriched, while in An04, subnets associated with intracellular trafficking, secretion, vesicular transport, chromatin structure, and dynamic Ca^2+^ signal transduction were established. The subnet of signal transduction mechanisms in Yugu2 was composed of a series of protein kinases and phosphatases. Proteins including calcium-dependent protein kinases and calcium-binding proteins constituted the Ca^2+^ signal transduction subnet in An04. The different signal pathway participation and interaction subnet construction indicated that the signal perception and response mechanisms were significantly different between different foxtail millet varieties.

### Functional Verification for the Phosphorylation of Sucrose Synthase (SuS) S10 Involved in Salt Tolerance

**FIGURE 7:**
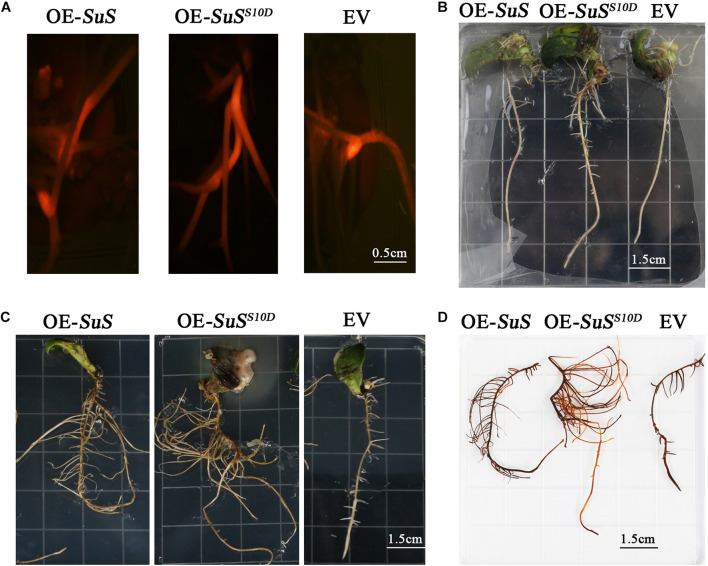
Effects of salt treatment on transgenic roots of soybean plants. **(A)** Identified transgenic roots with red fluorescence, OE-SuS: *SuS* over-expressing lines, OE-*SuS*^*S*10*D*^: over-expressing lines of *SuS*^*S*10*D*^, and EV: empty vector lines. **(B)** The phenotypes of the transgenic roots of OE-SuS, OE-*SuS*^*S*10*D*^and EV before salt treatment. **(C)** The phenotypes of the transgenic roots of OE-SuS, OE-*SuS*^*S*10*D*^ and EV after salt stress. **(D)** *In situ* detection of H_2_O_2_ by DAB staining of transgenic roots after salt stress.

Sucrose metabolism is tightly coupled with sugar signaling, cellulose and flavonoids biosynthesis (Ruan, 2014; Jia et al., 2019). The phosphorylation of three SuSs (K3XVC3, K4A5 × 5, K4A5Y9) were down regulated in phosphorylation at Ser in the N-terminus in An04, while these phosphorylated sites were significantly up-regulated in Yugu2 (Supplementary Table 13). To verify the function of phosphorylation sites in the identified DRPPs, the SuS (K3XVC3) that catalyzes the reversible cleavage of sucrose and plays a key role in sugar metabolism was selected. The phosphorylation level of site Ser10 in protein was down-regulated in An04, while this site was significantly up-regulated 2.5-fold in Yugu2. The phospho-mimic SuS^*S*10*D*^ mutant was created by changing the phosphorylation site Ser residues to Asp residues. The *SuS* and *SuS*^*S*10*D*^ were transformed into the cotyledonary node of Qihuang 34 soybean and the plants with transgenic roots were selected through RFP detection (Figure 7A). After treating with 150mM NaCl, the plants with transgenic roots expressing *SuS*^*S*10*D*^ showed higher tolerance than those transformed with *SuS* with significant increase in biomass. In contrast, the growth of roots transformed with empty vector (EV) were inhibited under this condition (Figures 7B,C). DAB (3,3′-diaminobenzidine) staining showed a greater accumulation of H_2_O_2_ in control roots than those in the transgenic lines with the *SuS*^*S*10*D*^-expressing roots, and accumulating less H_2_O_2_ compared to *SuS*-over-expressing roots (Figure 7D). This result indicated that the phosphorylation at S10 is required for the activation of SuS, and the phosphorylation of SuS might perform a vital role in the salt tolerance of foxtail millet.

## Discussion

Plant responses to salt stress are highly conserved and fine-tuned. Similar to response to other environmental stresses, this response is mediated by sophisticated molecular mechanisms among which reversible modification in protein phosphorylation play a pivotal role. Here, we constructed a holistic atlas of salt-responsive changes in proteomic and phosphoproteomics of foxtail millet at the onset of salt stress, and a large number of stress responsive proteins and phosphoproteins were identified.

### Ca^2+^-Depended Kinase Singling and ABA Signaling Were Critical for Salinity Perception and Transduction in Foxtail Millet

Salt stress is communicated by ion toxicity (mainly Na^+^) and osmotic stress signals in plants, which can activate the corresponding signal transduction pathways. Osmotic and ionic stresses trigger elevated cytosolic free calcium concentrations. Calcium-transporting ATPases (Ca^2+^ pumps) are thought to participate in a fine tuning of [Ca^2+^]_*cyt*_ signatures in response to environmental stimuli (Bonza et al., 2010; Bose et al., 2011). Expressing calcium transport ATPases (*AtACA4* and *AtACA2*) in *Saccharomyces cerevisiae* enhances salt tolerance (Bose et al., 2011). Ca^2+^ signatures are decoded by downstream effector proteins to generate specific biological responses (Zeng et al., 2015). These effectors include three major types of Ca^2+^ sensor proteins, calmodulin (CaM)/CaM-like (CML) proteins, calcineurin B-like (CBL) proteins, and calcium-dependent protein kinases (CDPK) (Zeng et al., 2015). During salt stress, the activated CDPK3 can phosphorylate and activate the vacuolar potassium channel TPK1, and thus sustain potassium efflux in order to maintain a high cytosolic K^+^/Na^+^ ratio (Latz et al., 2013). CaM/CaM-like proteins regulate a series of downstream targets including ion channels, protein kinases, phosphatases, transcription factors, metabolic enzymes, and other factors. The calmodulin-binding transcription activators (CAMTA) mediated gene transcription regulation is a key process for plants’ responses to abiotic stresses (Yue et al., 2015). Here, the phosphorylation of Ca^2+^ signaling related proteins was identified to be involved in salt stress response in foxtail millet (Supplementary Table 13). CAMTA (K4A595) was identified to be activated in salt-sensitive An04 and salt-tolerance Yugu2 cultivars. We observed an increase in phosphorylation of K3Z3F7, a calcium transport ATPase, in Yugu2, but a decrease in An04. Additionally, the phosphorylation of three CDPKs (K3XVS3, K3ZS81, and K3Y6F0) were increased in Yugu2, but decreased in An04 upon salt stress, and two other CDPKs (K3Y6A5 and K4A8A9) had higher phosphorylation levels in Yugu2 than those in An04 under salinity. The higher phosphorylation level of Ca^2+^ signaling related components in salt-tolerance Yugu2 indicated that this pathway may play a significance role in regulating salt tolerance in foxtail millet.

The ABA signaling pathway plays pivotal roles in plant adaptation to stress through regulation of various physiological processes. Recent reports have shown that ABA signaling components, such as PYR/PYL/RCAR receptors, 2C-type protein phosphatases (PP2C), SnRK2 (SNF1-RELATED PROTEIN KINASE 2) protein kinases, and ABA INSENSITIVE 5 (ABI5) were regulated via phosphorylation in response to abiotic stresses (Hu et al., 2015; Zhong et al., 2017; Zhao et al., 2019). The phosphorylation of ABI5 is required for its stability and activation of the expressions of stress adaptation genes (Skubacz et al., 2016). Consistent with these finding, the phosphorylation levels of the ABA receptor PYL2 (K3YVT2) and ABI5 (K3XJV0) were increased in both Yugu2 and An04 after salt treatment.

Phosphoinositides (PIs), the primary lipid-derived signals, are involved in various cellular processes, ranging from signal transduction to membrane trafficking and cytoskeleton organization (Heilmann, 2016). In response to abiotic stress, enzymes involved in PIs metabolism are also regulated by phosphorylation modification (Pi et al., 2017; Liu et al., 2019). In our research, phosphorylation levels of four enzymes (K3Z3H3, K3ZRE8, K3XVG6 and K3Y708) related to PI metabolism increased in Yugu2 upon salt treatment, while under the same condition in An04 only phosphoinositide phospholipase C (PI-PLC, K3Y708) was differentially phosphorylated (Supplementary Table 13). Salt and osmotic stress rapidly induce the PI-PLC activity, which further hydrolyzes phosphatidylinositol-4,5-bisphosphate (PIP_2_) into two secondary messengers: diacylglycerol (DAG) and Inositol 1,4,5-tris phosphate (IP_3_). This process eventually leads to the release of Ca^2+^ into the cytoplasm (Li et al., 2017). In *Arabidopsis*, AtPLC4 negatively regulates the salt stress response, and *plc4* mutant seedlings are hyposensitive to salt (Xia et al., 2017). In rice, OsPLC1 modulated Ca^2+^ signaling is essential for controlling Na^+^ accumulation in leaf, thus conferring plant salt tolerance (Li et al., 2017). The phosphorylated enzymes involved in PI metabolism may be responsible for governing the Ca^2+^ signaling and salt tolerance in foxtail millet. Phosphatidate phosphatase (PAP) catalyzes the dephosphorylation of phosphatidate to yield DAG, which then is used for the synthesis of phospholipids and triacylglycerol (TAG) (Pascual and Carman, 2013). The phosphorylation level of sites Tyr123 and Ser132 of PAP (K3Z3H3) were up regulated 1.4- and 1.3-fold, respectively, in Yugu2 than those of An04 after salt stress (Supplementary Table 13), which indicated the determinant role of phosphorylation modification in phosphoinositide metabolism, and the key effects of these phosphorylated sites in salt resistance of the varieties. These also consistent with our newly reported, the glycerophospholipid metabolism pathway was highly-enriched in Yugu2, and the pathway may play a decisive role in salt tolerance of foxtail millet (Pan et al., 2020).

In addition to Ca^2+^ and ABA signaling, a large number of serine/threonine-protein kinases, RLKs and phosphatases, which play pivotal roles in signal transduction, were also differentially phosphorylated under salinity. Among the differentially phosphorylated phosphoproteins, two kinases, namely K3YPG5 and K3YQ71, were responsive to salt stress in both varieties, while out of the remaining 19 protein kinases, 13 were phosphorylated and 6 were dephosphorylated only in Yugu2 under salinity. Seventeen protein kinases possess higher phosphorylation levels in Yugu2 compared with An04 after salt stress. Two mitogen-activated protein kinases (K3XXQ0 and K3XXF6) and two serine/threonine-protein phosphatases (K4A5B5 and K4A857) were also identified to be activated in Yugu2 under salinity (Supplementary Table 13). In An04, phosphorylation level of 4 kinases increased and phosphorylation level of 2 kinases decreased under salinity (Supplementary Table 13). In rice, receptor-like kinase OsSIK2 integrates stress signals into a developmental program for better adaptive growth under stress conditions and *OsSIK2* overexpressing plants demonstrated enhanced salt and drought resistance (Chen et al., 2013). In rice, salt stress rapidly activates lectin receptor-like kinase SIT1, which phosphorylate MPK3/6 and result in ethylene and ROS overproduction, thereby leading to growth inhibition and eventual plant death (Li et al., 2014). GUDK-mediated transphosphorylation of transcription factor OsAP37 is activated by drought stress and is required for transcriptional activation of stress-regulated genes that enhance tolerance and improve yield under stress (Ramegowda et al., 2014). Cold-activated protein kinase CRPK1 phosphorylates 14-3-3 proteins, which are required for transducing the cold signal from the plasma membrane to the nucleus and fine-tuning CBF-dependent cold response (Liu et al., 2017). These salt stress-activated protein kinases may be responsible for phosphorylation of overrepresented [SP], [TP], [RxxS], and [SxD] motifs identified in DRPPs (Figure 5 and Supplementary Figure 5), and function as molecular mediators to integrate stress signals into cellular adaptive responses. The Ca^2+^, phytohormone, and phosphoinositide invoke signaling pathways for salt perception. The large number of protein kinases identified in Yugu2, combined with much higher levels of phosphorylation modification after NaCl treatment (Supplementary Table 13) indicated rapid and strong activation of salt stress perception and signal transduction which leading to a more intense response in Yugu2 compared with An04.

### Salinity Disturbed Protein Phosphorylation Are Involved in Modulating the Chromatin Remodeling, Transcription, and PTM

Under salt stress, many responses and defense-related genes are stimulated by upstream complex regulatory processes. These regulatory mechanisms include several different levels, such as chromatin remodeling, transcriptional regulation, pre-RNA processing, and translational processes. The DRPPs related to these processes were identified in foxtail millet under salt stress, and the related proteins were also modulated by phosphorylation (Supplementary Table 13).

Chromatin is the stabilized and condensed DNA. Its status can be modified by environmental or developmental stimuli and determine the accessibility and effectiveness of the transcriptional machinery (Kim et al., 2010; Asensi-Fabado et al., 2017). The switch (SWI)/sucrose non-fermenting (SNF) complex is a multi-subunit DNA-dependent ATPase that alters chromatin structure and contribute to transcriptional activation and RNA Polymerase II elongation (Schwabish and Struhl, 2007; Kim et al., 2010). In *Arabidopsis*, *swi3b* mutants (the core subunit of the SWI/SNF complex) showed reduced ABA sensitivity and restrained expression of the ABA-responsive genes (Kim et al., 2010). Therefore, chromatin remodeling is a potential means to regulate gene expression (Schwabish and Struhl, 2007; Kim et al., 2010). Changes in chromatin structure are associated with modification of the N-terminal tails of histones (Kim et al., 2010). The histones could be modified by methylation, acetylation, ubiquitination, and phosphorylation at different residues. The methylation and acetylation modification have been found to be influenced by drought and salinity stress and ABA treatment, but the phosphorylation of histones is not well characterized. Three residues of the histone H1 (K3ZW07), Ser191, Ser236, and Ser259, were strongly phosphorylated in An04. Salt stress raised the phosphorylation level of SWI/SNF complex subunit’s (K4A5I6) at the residue Ser479 in An04, but suppressed it in Yugu2 (Supplementary Table 13). The changes in the phosphorylation levels of histone H1 and the SWI/SNF complex subunit identified in this work indicated that chromatin structure remodeling might participate in salt stress response via activated stress related gene expression in foxtail millet.

In response to external stress, protein kinases or phosphatases change the phosphorylation status of transcription factors (TFs). Seven transcription factors were phosphorylated in An04 upon salt stress and one was dephosphorylated. In Yugu2, phosphorylation status of five transcription factors were significantly repressed. The phosphorylation level of the WRKY (K3ZW13) and the trihelix transcription factor (K3XK15) increased in An04, but decreased in Yugu2 (Supplementary Table 13). A large number of WRKY TFs are found to associate with salt stress, and the phosphorylation of WRKY72 has been related to enhanced salinity stress tolerance in rice (Phukan et al., 2016). WRKY could be phosphorylated and activated by kinases, such as MAPK and CDPK. This is an important regulatory mechanism that can be targeted for controlling expression of certain TFs (Phukan et al., 2016). Also our results may seem contradictory to previous findings, we believe that other likely mechanisms could regulate the expression of the stress-responsive genes. As stated above, our results also showed that the DRPPs related to chromatin remodeling and transcription, which may contribute to the activation of stress response gene expression, showed higher phosphorylation levels in An04 compared with Yugu2 after stress. These results are consistent with our previous results that fewer genes were induced in salt-tolerance cultivar Yugu2 compared with salt-sensitive cultivar An04 under salinity (Pan et al., 2020). Numerous genes related with salt responsive, detoxification and ion channels are constitutively expressed at higher levels in Yugu2, which may also be key to its salt resistance.

Normally, severe stresses could dramatically decrease the overall translation rates (Merchante et al., 2017). However, the mRNAs coding for proteins that play a key role in plant survival are found to be actively translated. In most cases, translation regulation allows for translation of specific sets of transcripts (Muench et al., 2012). The phosphorylation status of specific ribosomal proteins (RP), initiation factors (eIFs), and poly(A)-binding proteins (PABP) regulate translation initiation (Muench et al., 2012). Developmental cues and stress cues trigger phosphorylation of RP, specific eIFs, and PABP, thereby confer drastic effects on translation, either by inhibiting or activating translation globally (Merchante et al., 2017). After salt stress, the phosphorylation status of ribosomal proteins were altered in both Yugu2 and An04. Five translation initiation factors were phosphorylated in Yugu2, while only one (K3Z381) was phosphorylated in An04 after stress (Supplementary Table 13). The TOR signaling pathway regulates ribosomal protein S6 (RPS6) phosphorylation, which is involved in selective translation of mRNAs in plants (Merchante et al., 2017). Under submergence, *Arabidopsis* SnRK1 phosphorylates the translation initiation factor eIFiso4G to enhance the translation of specific mRNAs, which include core hypoxia response genes and genes related to stress response and biosynthetic processes (Cho et al., 2019). RP and eIF phosphorylation can contribute to the repression or initiation of the translation of a subset of mRNAs. The four specific phosphorylated eIFs in Yugu2 (K3Y5B8, K3Z4I7, K4A6E4, K3YSD3) might trigger translation of certain mRNAs that are critical for salt tolerance.

### The Phosphorylation of Proteins Involved in Ion and Water Transport Were Modified

In response to excess ions, it is important to maintain cellular ion homeostasis via reducing cytoplasmic Na^+^ and increasing cytoplasmic K^+^. Na^+^-triggered Ca^2+^ signals are sensed by SOS3 and activated by SOS2, which in turn phosphorylates and activates the Na^+^/H^+^ antiporter SOS1. The activated SOS1 is responsible for transporting Na^+^ from the cytoplasm to the apoplast, which is driven by the proton gradient established by the plasma membrane H^+^-ATPase (PM H^+^-ATPase) (Yang and Guo, 2018). The SOS1 homolog in foxtail millet, K3Z3A4, was phosphorylated in both Yugu2 and An04 during the response to salinity. In parallel, the phosphorylation level of a plasma membrane ATPase, K3ZQF6, was markedly enhanced in Yugu2, but repressed in An04. Two potassium transporters, namely K3Y5D3 and K3YD82 showed lower phosphorylation in An04 (Supplementary Table 13). These results are in line with our previous reports that Yugu2 is more effective in ion homeostasis regulation (Pan et al., 2020).

Aquaporins (PIP) act as multifunctional channels responsible for the transport of water, some small neutral solutes, and ions. Root hydraulic conductivity is positively correlated with PIP phosphorylation, and phosphorylated Ser in the C-terminal of aquaporins can activate the water channel, which can be inactivated through dephosphorylation (McGaughey et al., 2018). There are many reports indicating that the phosphorylation of PIPs were altered in response to salinity in *Brachypodium distachyon*, sugar beet, *Arabidopsis*, and other plants (McGaughey et al., 2018). This modification in PIP phosphorylation can be part of adaptation to osmotic and ionic stresses via adjusting PIP Na^+^ transport. Phosphorylation level of the C-terminus Ser of four aquaporins, K3YUP4, K3Y958, K3ZVT1, K3ZVT2, decreased under salt stress in An04, but there were no obvious changes in phosphorylation status of these aquaporins in Yugu2 (Supplementary Table 13). This dephosphorylation of PIP in AN04 might cause a switch-off of the water channel in the plasma membrane, which may partially explain the previously reported drought sensitivity of An04 (Tang et al., 2017). The *Arabidopsis* sucrose-induced receptor kinase, SIRK1, directly interacts with and activates aquaporins via phosphorylation during sucrose-specific osmotic responses (Wu et al., 2013). Leaf panicle 2 (LP2), a leucine-rich repeat (LRR)-RLK, interact with three drought-responsive aquaporin proteins, indicating that these aquaporins may be phosphorylation targets of LP2 (Wu et al., 2015). In rice, aquaporins OsPIP2;1/OsPIP2;6 are phosphorylated by OsCPK17 in a calcium-dependent manner in response to cold stress (Almadanim et al., 2017). The alteration of phosphorylation levels of the aquaporins identified in our results may be the target of activated RLKs, CDPK and phosphatases in response to salinity.

Recently, it is shown that vesicular trafficking and endocytosis play vital role during adaptation of plants to salt stress. Vesicular trafficking is the concerted process that starts with the budding of transport vesicles from donor membranes and ends with their fusion to target organelles. It is presumed that endocytosis removes transporters and channels from the plasma membrane (PM) to limit Na^+^ entry into the cell and internalize PM aquaporins to block water loss (Baral et al., 2015). Endosomal vesicles were suggested to contain NHX isoforms, which could be sequestering the excess cytosolic Na^+^ within the vesicles that subsequently fuse to the vacuole, thus helping to modulate the cytosolic Na^+^ concentration (Bassil et al., 2012). ADP-ribosylation factor GTPase-activating proteins (ARF-GAP) can mediate membrane trafficking and actin remodeling via regulating the activity of ADP-ribosylation factor GTPase (ARF-GTPases), which function in vesicle formation and dissociation (Du et al., 2011; Takacs et al., 2012). An increase in ARF-GAPs K3XEU4 and K3YQD1 phosphorylation were observed in both An04 and Yugu2 cultivars in response to salt stress. However, in Yugu2 K4AKS6, an additional ARF-GAP was subject to increase in phosphorylation (Supplementary Table 13). Overexpression of ARF-GAP gene *OsAGAP* in rice stimulates vesicle transport from the plasma membrane to the Golgi apparatus (Du et al., 2011). ARF-GAP in *Arabidopsis* is known to function in signaling pathways with PIs to facilitate rebuilding of the actin cytoskeleton, and directional membrane trafficking during root hair development (Yoo et al., 2012). The activated ARF-GAPs in this work may contribute to the promotion of vesicular trafficking to enhance salt tolerance and control root development during salt stress in foxtail millet.

### Phosphorylation of Proteins Involved in Sucrose Metabolism Play a Significant Role in Salt Response in Foxtail Millet

Sucrose is the main product of photosynthesis and its metabolism yields hexoses that are necessary to generate energy and synthesize cellulose, starch, and antioxidants. Sucrose also functions as a signaling molecule to regulate many vital metabolic processes and activate stress response pathways (Ruan, 2014). Sucrose-phosphate synthase (SPS) and SuS are the primary enzymes involved in sucrose synthesis and cleavage. SPS is responsible for the irreversible catalysis of UDP-glucose and fructose-6-phosphate into sucrose-6-phosphate, while SuS catalyzes the reversible conversion of sucrose and UDP into fructose and UDP-glucose (Huang et al., 2016). In this work, the phosphorylation of foxtail millet SPS, K3YG05, increased in both An04 and Yugu2. Salt stress reduced the phosphorylation of three SuSs, namely K3XVC3, K4A5 × 5, and K4A5Y9, at the Ser residue in the N-terminus in An04. In contrast, these genes were increased phosphorylation in Yugu2 (Supplementary Table 13). SPS is differentially activated or deactivated by protein phosphorylation under abiotic stress, probably in different serine residues (Ruan, 2014). In the early stage of cold stress, OsCPK17 phosphorylates OsSPS4 and reduces its activity, ultimately affected sugar metabolism (Almadanim et al., 2017). SuS from diverse plant tissues is phosphorylated by CDPK at a conserved Ser residue located near its N-terminus. This modification enhances the sucrose degradation that may provide UDP-glucose for the cellulose or callose synthesis and energy production (Takeda et al., 2017). The two proteins involved in glycolysis, 6-phosphofructokinase, K3ZDH8, and fructose-bisphosphate aldolase, K3YTL8, also showed enhanced phosphorylation in Yugu2 under salt stress. Our previous research demonstrated that the enzymes related to glycolysis and the TCA cycle are significantly up regulated in response to drought. This is expected to enhance carbon metabolism and the energy supply for stress tolerance (Pan et al., 2018). We also showed that in response to salt stress, the contents of UDP-glucose and fructose 6-phosphate decreased in the Yugu2 roots (Pan et al., 2020).

Sucrose metabolism is tightly coupled with sugar signaling, which is achieved by production of sugar signaling molecules including sucrose, glucose (Glc), fructose (Fru), and trehalose-6-phosphate or perhaps by the signaling role applied by the metabolic process itself (Ruan, 2014). In *Arabidopsis*, sucrose signaling induces flavonoids biosynthesis by activating the expression of MYBL2. In *Malus halliana*, sucrose could act as a signaling molecule to regulate ROS homeostasis through inducing flavonoid synthesis and alkaloid metabolism under saline–alkali stress (Jia et al., 2019). Additionally, our recent research showed that a large number of flavonoids, including flavones, flavonols, flavanones, and anthocyanins accumulate in Yugu2 root during salt stress, and the genes encoding key enzymes related to phenylpropanoid and flavonoid biosynthetic pathways were up-regulated (Pan et al., 2020). The phosphorylation of two phenylalanine ammonia-lyases (PAL), K3YQC4 and K3YGI0, also increased in Yugu2 after salt stress (Supplementary Table 13). PAL is the first committed enzyme in phenylpropanoid and flavonoid biosynthetic pathways, which catalyzes the conversion of phenylalanine to cinnamic acid (Saito et al., 2013). The phosphorylated PAL may be responsible for launching flavonoid biosynthesis by providing the related precursor. It could then be deduced that the activated flavonoid synthesis pathways might be mediated by sucrose signaling in salt stress response of foxtail millet.

Cellulose is the dominant load-bearing polysaccharide of plant cell walls. The catalytic activity of cellulose synthase A (CESA) is dynamically regulated by phosphorylation in response to a variety of developmental and environmental stimuli (Speicher et al., 2018). SuS also takes part in cell wall biosynthesis by supplying UDP-glucose, the substrate for biosynthesis of cellulose (Ruan, 2014). A cellulose synthase (K3ZQ90) was dephosphorylated in both Yugu2 and An04 upon salt stress, while the phosphorylation increased in K4A562, another cellulose synthase in An04 (Supplementary Table 13). The phosphorylated sites within CESA might potentially govern CESA–microtubule association and overall crystalline cellulose production (Speicher et al., 2018). Abiotic stresses, including salt stress, can alter cell-wall deposition and remodeling, and this is monitored by cell-wall-integrity (CWI)-sensing protein kinases (Speicher et al., 2018). Increasing evidence indicate that the RLKs, such as THE1, MIK2, FER, FEI1, FEI2, and wall-associated kinases (WAK) are involved in sensing and relaying salt stress signals via monitoring CWI, and might function as direct or indirect regulators of cellulose biosynthesis (Zhao et al., 2020). Our results of differential modulation of cellulose synthases in foxtail millet in response to salinity were consistent with recent reports, that demonstrating the cell wall damage and component change upon salt stress are possible salt-sensing mechanisms that further activate salt responses and avoidance mechanisms, and regulate root architecture (Jia et al., 2019; Zhao et al., 2020). Thus, regulation of sucrose metabolism may play significant roles in salt response in foxtail millet by enhancing energy production, inducing flavonoid synthesis, and modulating cellulose biosynthesis.

## Conclusion and Perspectives

**FIGURE 8:**
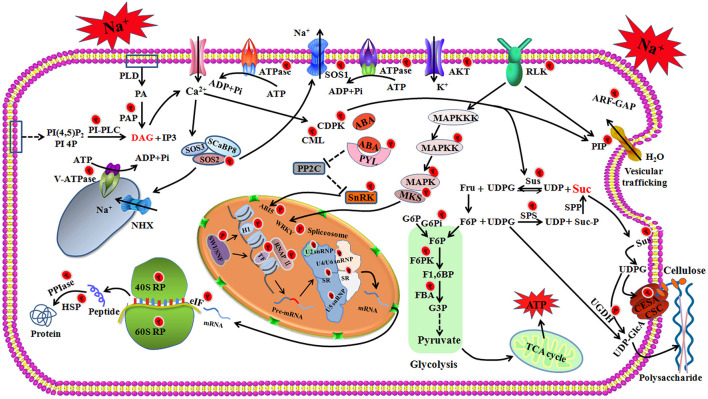
The schematic presentation of salt-responsive mechanisms in the root of foxtail millet. RLK, receptor-like protein kinase; SOS, salt overly sensitive; SCaBP8, SOS3-Like calcium binding protein 8; AKT, potassium transporter; MAPK, Mitogen-activated protein kinase; MAPKK, Mitogen-activated protein kinase kinase; MAPKKK, Mitogen-activated protein kinase kinase kinase; PP2C, protein phosphatase 2C; PYL, abscisic acid receptor; PLD, phospholipase D; PA, Phosphatidic acid; PAP, phosphatidate phosphatase; DAG, Diacylglycerol; PI(4,5)P2, phosphatidylinositol 4,5-bisphosphate; PI4P, phosphatidylinositol-4-phosphate; IP3, inositol 1,4,5-trisphosphate; PI-PLC, Phosphoinositide phospholipase C; hnRNPs, Heterogeneous nuclear ribonucleoproteins (hnRNPs); snRNPs, Small nuclear ribonucleoproteins (snRNPs); SR, serine/arginine-rich splicing factor; RP, ribosomal protein; eIF, Eukaryotic translation initiation factor; PPIase, peptidyl-prolyl cis-trans isomerase; HSP, heat shock protein; SuS, Sucrose synthase; SPS, Sucrose phosphate synthase; SPP, sucrose phosphate phosphatase; Suc, Sucrose; Suc-P, Sucrose 6-phosphate; Fru, Fructose; UDPG, Uridine diphosphate glucose; UDP, uridine diphosphate; F6P, fructose 6-phophate; F1,6BP, fructose 1,6-bisphosphate; G6Pi, Glucose-6-phosphate isomerase; G6P, Glucose-6-phosphate; FBA, Fructose-bisphosphate aldolase; F6PK, ATP-dependent 6-phosphofructo kinase; CSC, cellulose synthase complex; CESA, cellulose synthase A; UGDH, UDP-glucose dehydrogenase; UDP-GlcA, UDP-glucuronate.

The results obtained by a global survey of quantitative proteomics and phosphoproteomics have yielded valuable information about the response of foxtail millet to salt stress. Upon salt stress, the excess sodium triggers calcium signal, and the SOS signaling, ABA signaling, phosphatidic acid (PA) signaling, and MAPK cascades to relay the signal downstream. The expressions of salt-responsive genes are regulated at several different levels, including chromatin remodeling, transcriptional regulation, RNA processing, translational processes, and post-translational modification through phosphorylation or dephosphorylation (Figure 8). The activated SOS pathway, K^+^ channels, aquaporins and vesicular trafficking contribute to the maintenance of cellular ion homeostasis. CDPK phosphorylated SPS and SuS contribute to sucrose degradation, which provides UDP-glucose for the cellulose or callose synthesis and energy production. Sucrose signaling may also induce flavonoid synthesis to control the ROS homeostasis under salt stress (Figure 8). Additionally, salt stress alters the cell wall by regulating the phosphorylation of cellulose synthase. This may be another salt-sensing mechanism to further activate plant salt response and avoidance mechanisms. Together, our research presented a phosphoproteomic landscape in the fine-tuning regulation network of foxtail millet in response to salt stress. The results described here will be a springboard for further investigation of stress adaptation mechanisms of foxtail millet and salt resistance breeding initiatives.

## Data Availability Statement

The datasets presented in this study can be found in online repositories. The names of the repository/repositories and accession number(s) can be found in the article/Supplementary Material.

## Author Contributions

JP and WL conceived and designed the research. ZL and QW carried out the experiments. JP, ZL, YG, XL, YH, FM, JL, and SD carried out the bioinformatic analysis. JP, SD, and WL analyzed the results and wrote the manuscript. All authors contributed to the article and approved the submitted version.

## Conflict of Interest

The authors declare that the research was conducted in the absence of any commercial or financial relationships that could be construed as a potential conflict of interest.

## Publisher’s Note

All claims expressed in this article are solely those of the authors and do not necessarily represent those of their affiliated organizations, or those of the publisher, the editors and the reviewers. Any product that may be evaluated in this article, or claim that may be made by its manufacturer, is not guaranteed or endorsed by the publisher.

## Footnotes

1
http://www.uniprot.org/taxonomy/4555

2
http://geneontology.org/

3
http://www.ncbi.nlm.nih.gov/COG

4
http://www.genome.ad.jp/kegg/

5
http://www.genscript.com/wolf-psort.html

6
http://string-db.org

## Supplementary Material

Supplementary Figure 1 Principal component analysis (PCA) distribution of all samples using quantified proteins.

Supplementary Figure 2 **(A)** The clusters of orthologous groups of proteins’ (KOG/COG) classification of the DAPs in different comparison groups. **(B)** The subcellular localization of DAPs in different comparison groups.

Supplementary Figure 3 **(A)** The clusters of orthologous groups of proteins (KOG/COG) classification of the DRPPs between Yugu2 and An04 varieties before (YG2CvsAN04C) and after (YG2TvsAN04T) salt stress. **(B)** The subcellular localization of DRPPs between these two varieties before (YG2CvsAN04C) and after (YG2TvsAN04T) salt stress.

Supplementary Figure 4 GO functional cluster of DAPs in the biological process.

Supplementary Figure 5 Hierarchical clustering **(A)** and K-means clustering **(B)** of DAPs in Yugu2 and An04.

Supplementary Figure 6 K-means clustering and phosphorylation motif analysis of phosphorylation sites in Yugu2 and An04 under salinity.

Supplementary Figure 7 Protein–protein interaction networks of DRPPs in six functional categories of An04.

Supplementary Table 1 The identified proteins and phosphoproteins in Yugu2 and An04. This table include the information of Protein ID, protein peptides, protein abundance, matched modified sequences, abundance of phospshopeptides, normalized ratio, *p*-value and so on.

Supplementary Table 2 The statistics and list of differentially abundant proteins (DAPs) in four comparison groups, AN04TvsAN04C, YG2CvsAN04C, YG2TvsAN04T, and YG2TvsYG2C.

Supplementary Table 3 The statistics and list of differentially regulated phosphoproteins (DRPPs) in four comparison groups, AN04TvsAN04C, YG2CvsAN04C, YG2TvsAN04T, and YG2TvsYG2C.

Supplementary Table 4 KOG and subcellular location statistics and list of DAPs in four comparison groups, AN04TvsAN04C, YG2CvsAN04C, YG2TvsAN04T, and YG2TvsYG2C.

Supplementary Table 5 KOG and subcellular location statistics and list of DRPPs in four comparison groups, AN04TvsAN04C, YG2CvsAN04C, YG2TvsAN04T, and YG2TvsYG2C.

Supplementary Table 6 GO functional cluster statistics and list of DAPs in the biological process in four comparison groups, AN04TvsAN04C, YG2CvsAN04C, YG2TvsAN04T, and YG2TvsYG2C.

Supplementary Table 7 GO functional cluster statistics and list of DRPPs in the biological process in four comparison groups, AN04TvsAN04C, YG2CvsAN04C, YG2TvsAN04T, and YG2TvsYG2C.

Supplementary Table 8 List of eight clusters in K-means analysis of DAPs.

Supplementary Table 9 List of eight clusters in K-means analysis of DRPPs.

Supplementary Table 10 Phosphorylation motif analysis of phosphorylation sites in eight clusters in K-means analysis of DRPPs.

Supplementary Table 11 List of salt stress regulates the phosphorylation sites on kinases in four comparison groups, AN04TvsAN04C, YG2CvsAN04C, YG2TvsAN04T, and YG2TvsYG2C.

Supplementary Table 12 List of DRPPs involved in protein-protein interaction network in Yugu2 and AN04.

Supplementary Table 13 List of key DRPPs involved in salt response in the foxtail millet.

## Abbreviations

CBLs: calcineurin B-like protein
CDPK: calcium-dependent protein kinases
CDK: cyclin-dependent kinase
CIPK: CBL-interacting protein kinases
CK-II: casein kinase-II
CESA: cellulose synthase A
DAP: differentially abundant proteins
DRPP: differentially regulated phosphoproteins
DAG: Diacylglycerol
eIF: Eukaryotic translation initiation factor
hnRNPs: Heterogeneous nuclear ribonucleoproteins (hnRNPs)
HSP: heat shock protein
IP3: inositol 1,4,5-trisphosphate
MPK: Mitogen-activated protein kinase
MAPKK: Mitogen-activated protein kinase kinase
PA: Phosphatidic acid
PAP: phosphatidate phosphatase
PI-PLC: Phosphoinositide phospholipase C
PI: Phosphoinositides
PIP: Aquaporin
PLD: phospholipase D
PP2C: protein phosphatase 2C
PYL: abscisic acid receptor
RLK: receptor-like kinase
RP: ribosomal proteins
SAM: S-adenosylmethionine
SPS: Sucrose-phosphate synthase
SuS: sucrose synthase
SR: serine/arginine-rich splicing factor.
