# Supplementary figures and images for: Phosphoproteomic Profiling Reveals Early Salt-Responsive Mechanisms in Two Foxtail Millet Cultivars
Source: Front Plant Sci. 2021 Sep 20;12:712257. doi: 10.3389/fpls.2021.712257 (PMC8488109; doi:10.3389/fpls.2021.712257)

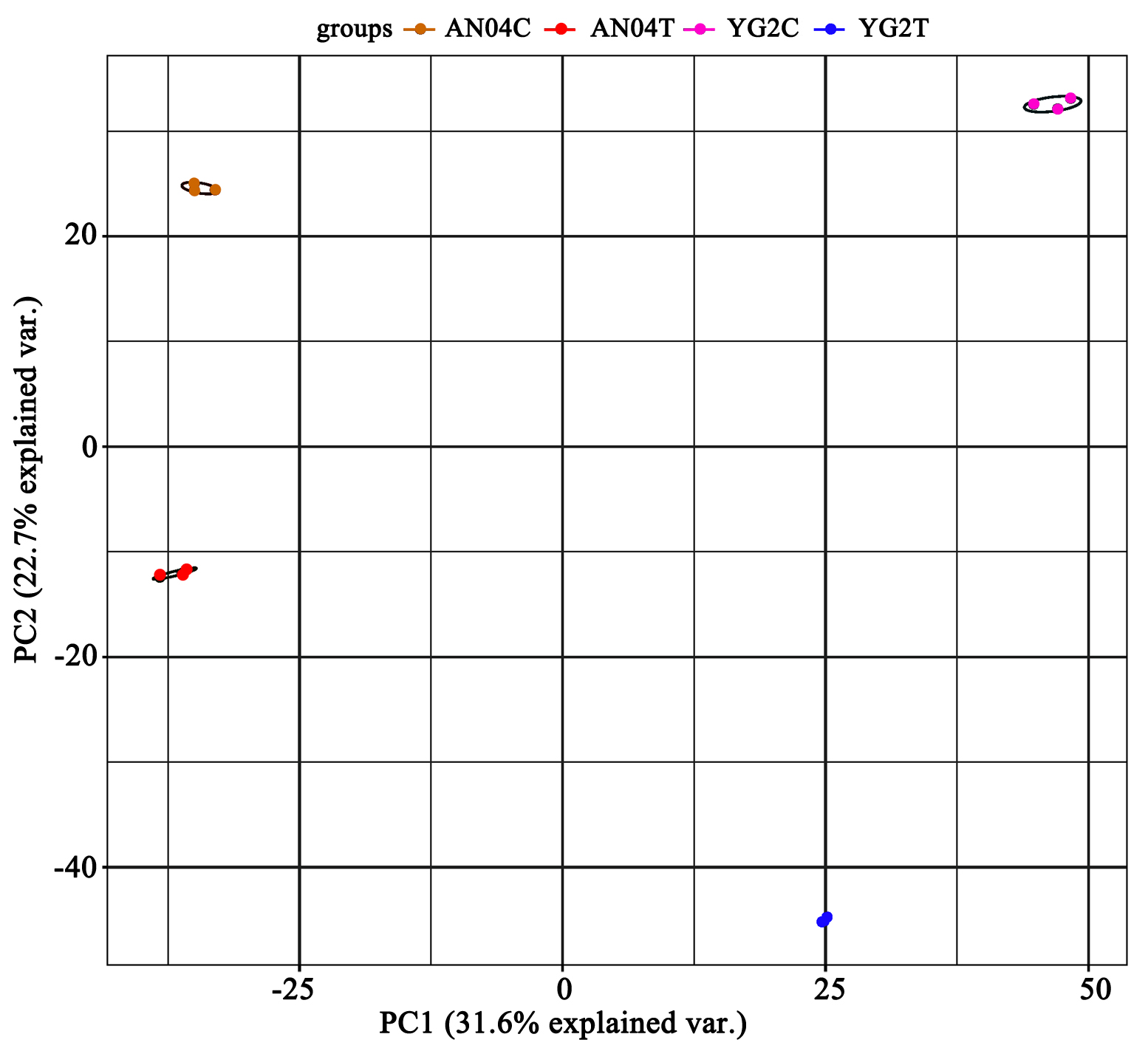

Supplement: Supplementary Figure 1 — Principal component analysis (PCA) distribution of all samples using quantified proteins. [file Image_1.JPEG]

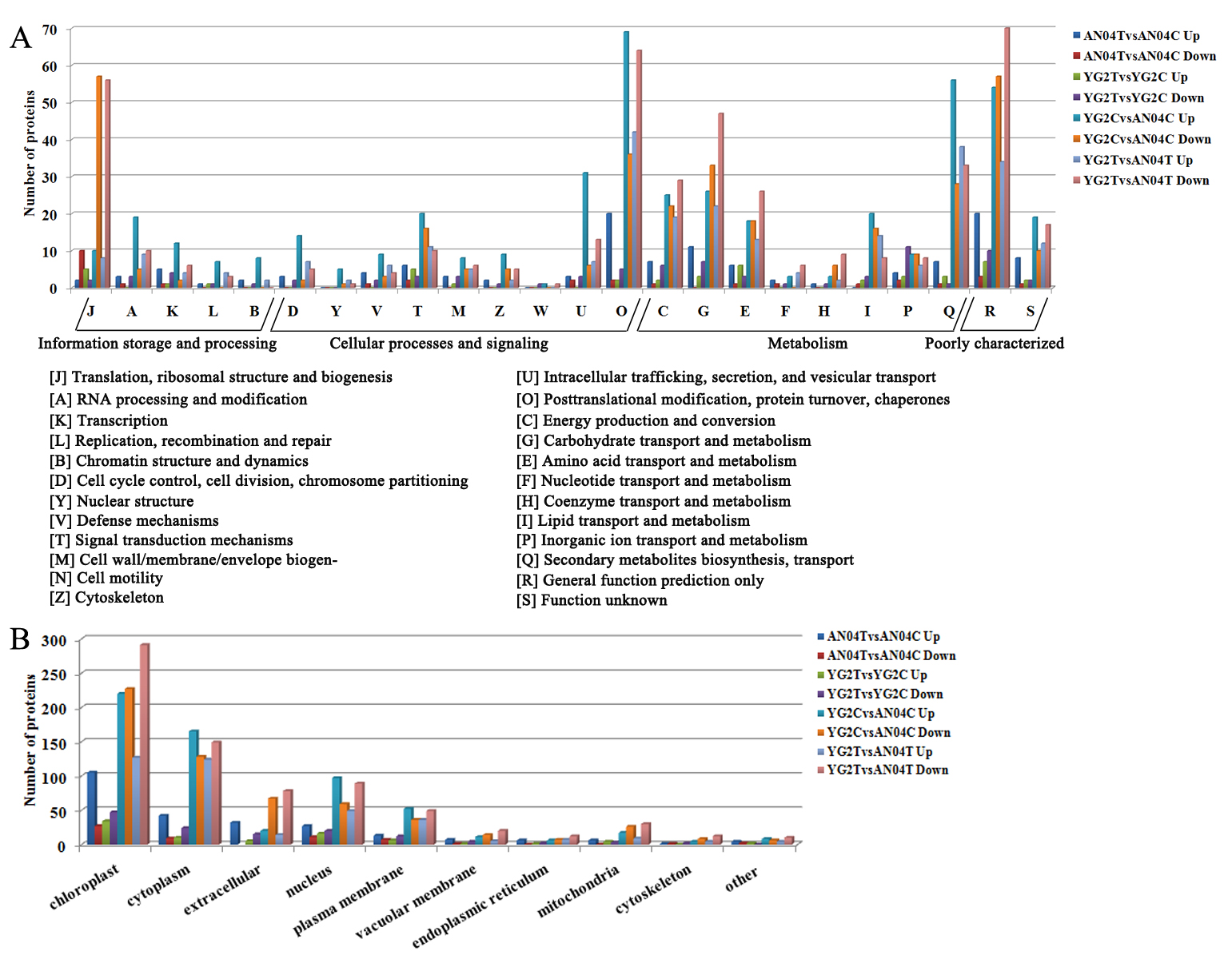

Supplement: Supplementary Figure 2 — (A) The clusters of orthologous groups of proteins’ (KOG/COG) classification of the DAPs in different comparison groups. (B) The subcellular localization of DAPs in different comparison groups. [file Image_2.JPEG]

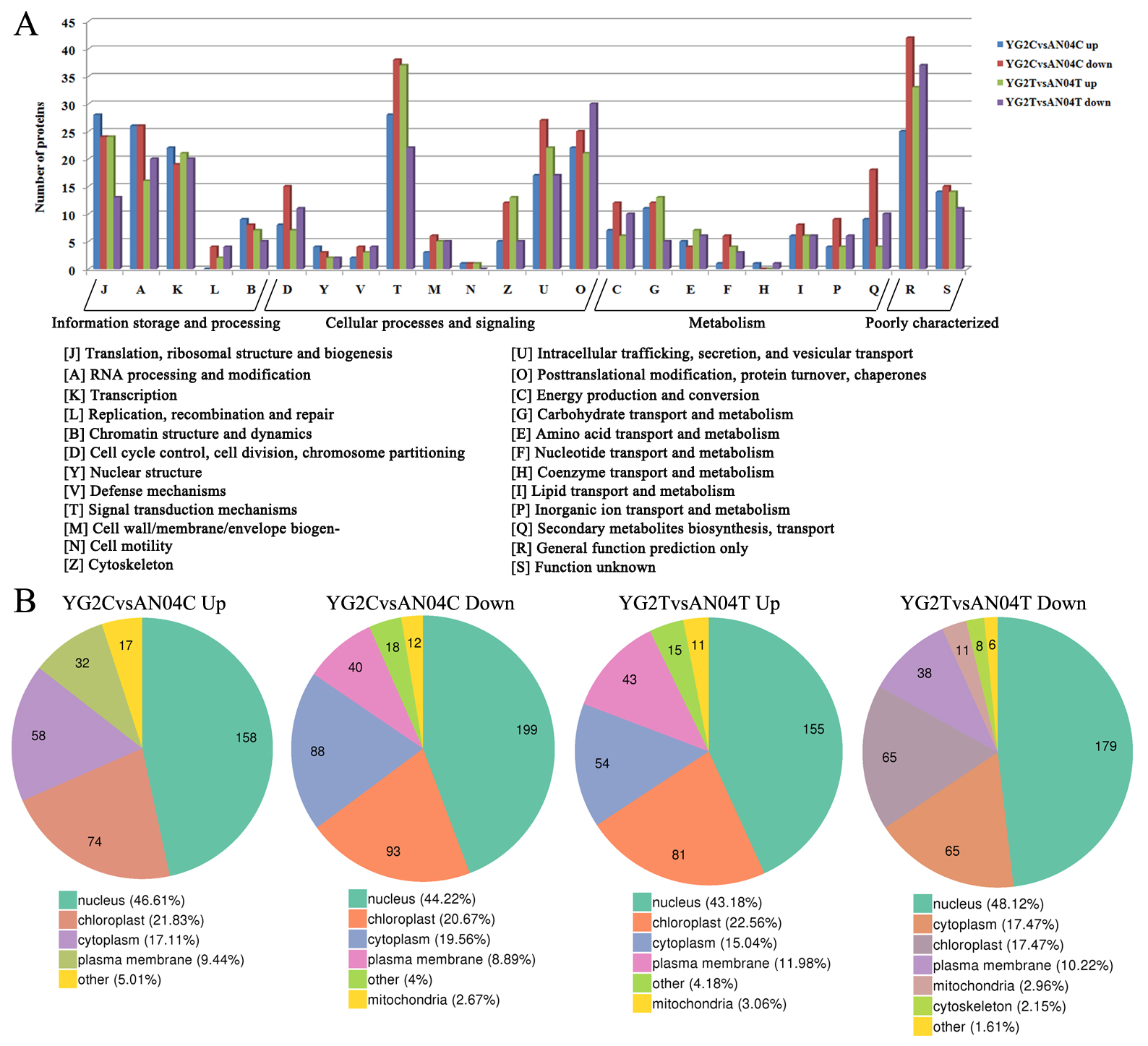

Supplement: Supplementary Figure 3 — (A) The clusters of orthologous groups of proteins (KOG/COG) classification of the DRPPs between Yugu2 and An04 varieties before (YG2CvsAN04C) and after (YG2TvsAN04T) salt stress. (B) The subcellular localization of DRPPs between these two varieties before (YG2CvsAN04C) and after (YG2TvsAN04T) salt stress. [file Image_3.JPEG]

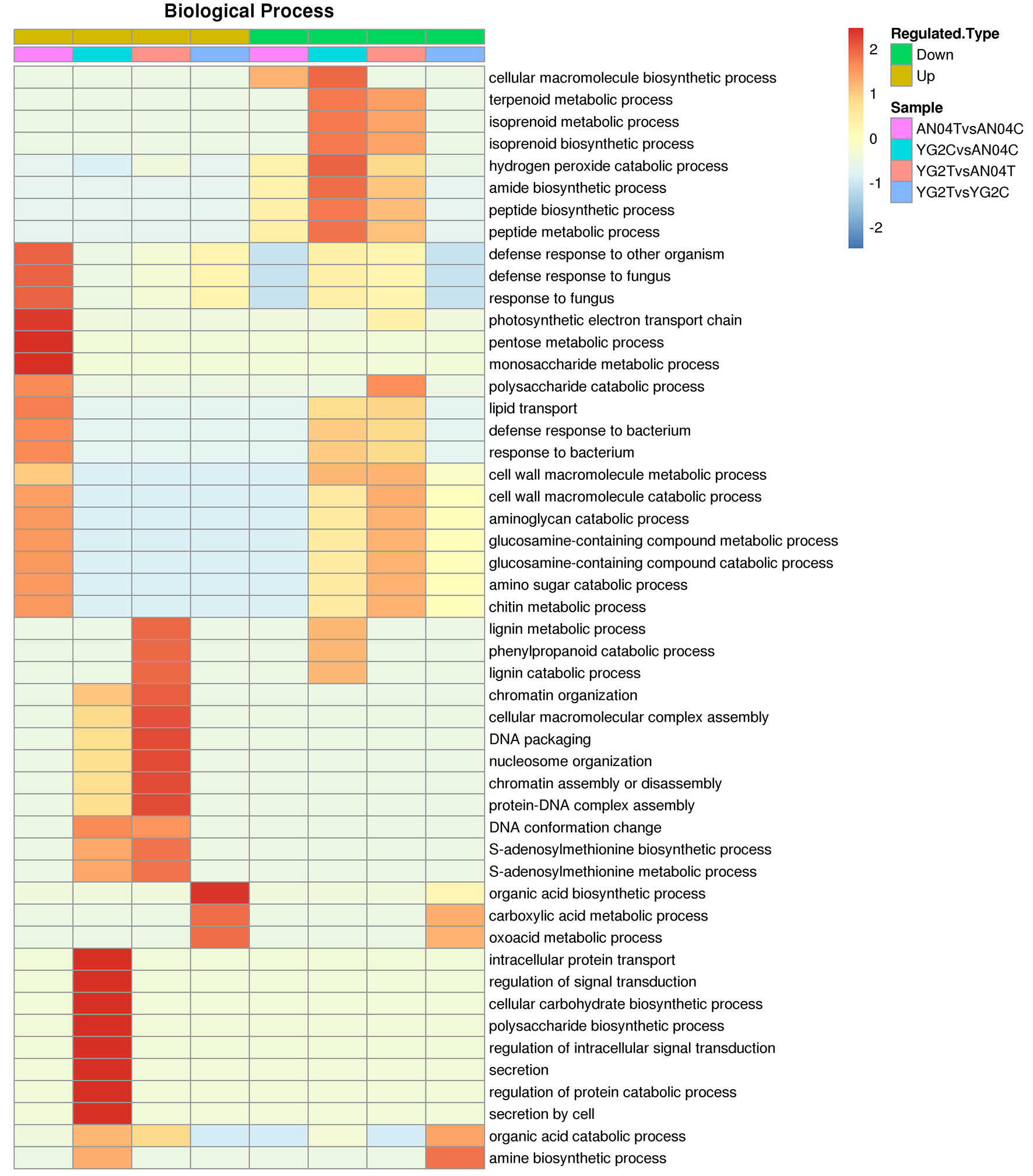

Supplement: Supplementary Figure 4 — GO functional cluster of DAPs in the biological process. [file Image_4.JPEG]

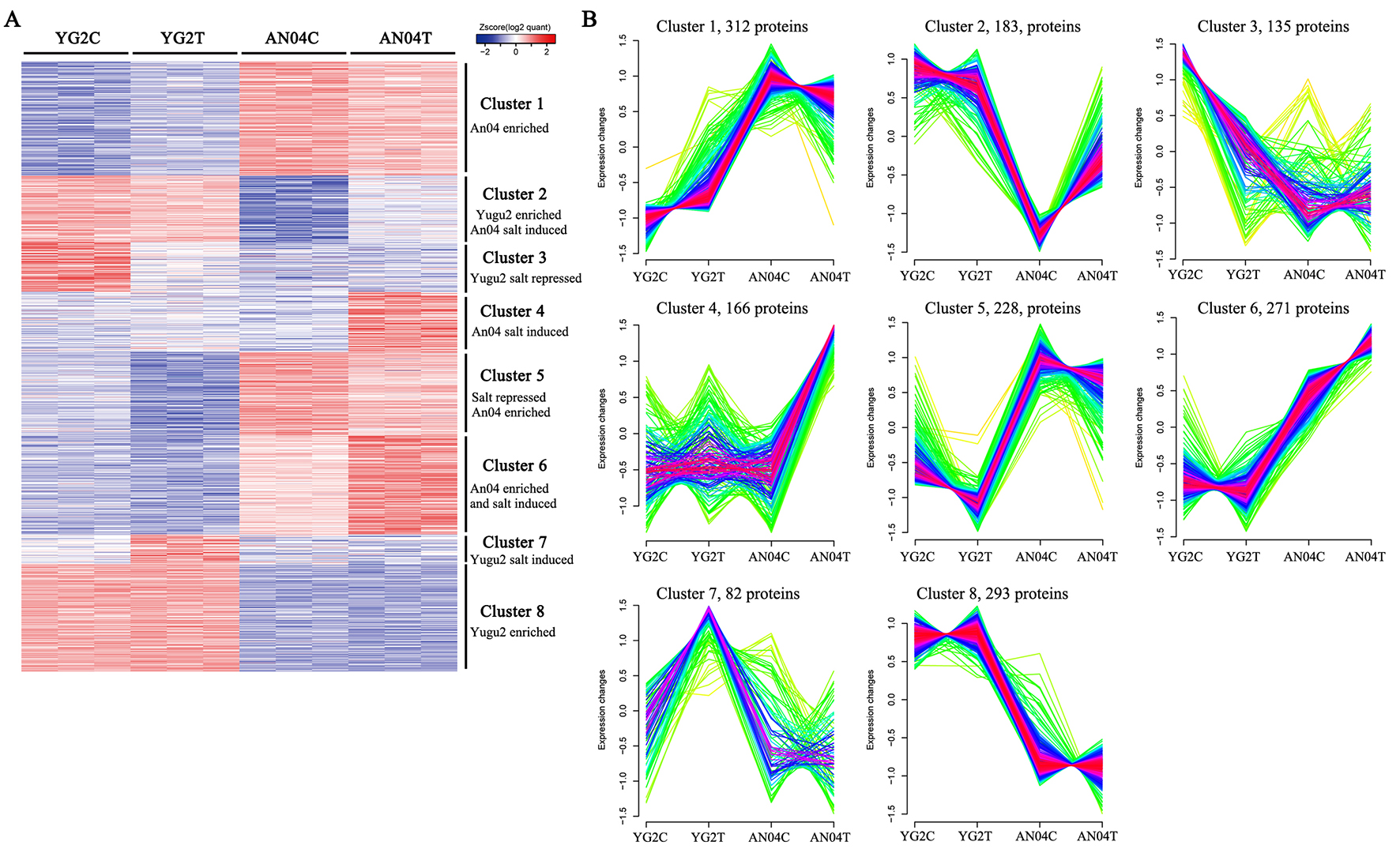

Supplement: Supplementary Figure 5 — Hierarchical clustering (A) and K-means clustering (B) of DAPs in Yugu2 and An04. [file Image_5.JPEG]

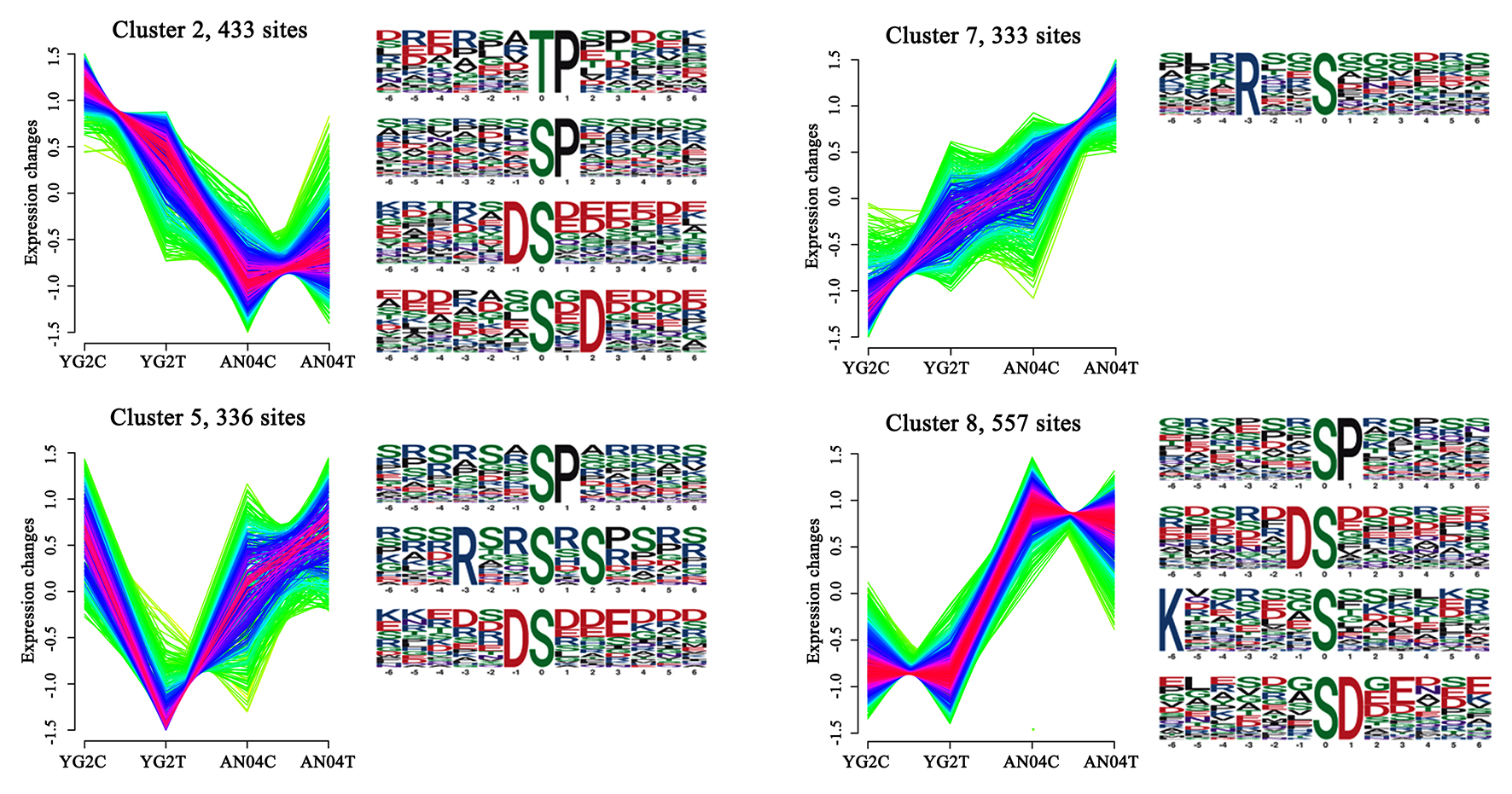

Supplement: Supplementary Figure 6 — K-means clustering and phosphorylation motif analysis of phosphorylation sites in Yugu2 and An04 under salinity. [file Image_6.JPEG]

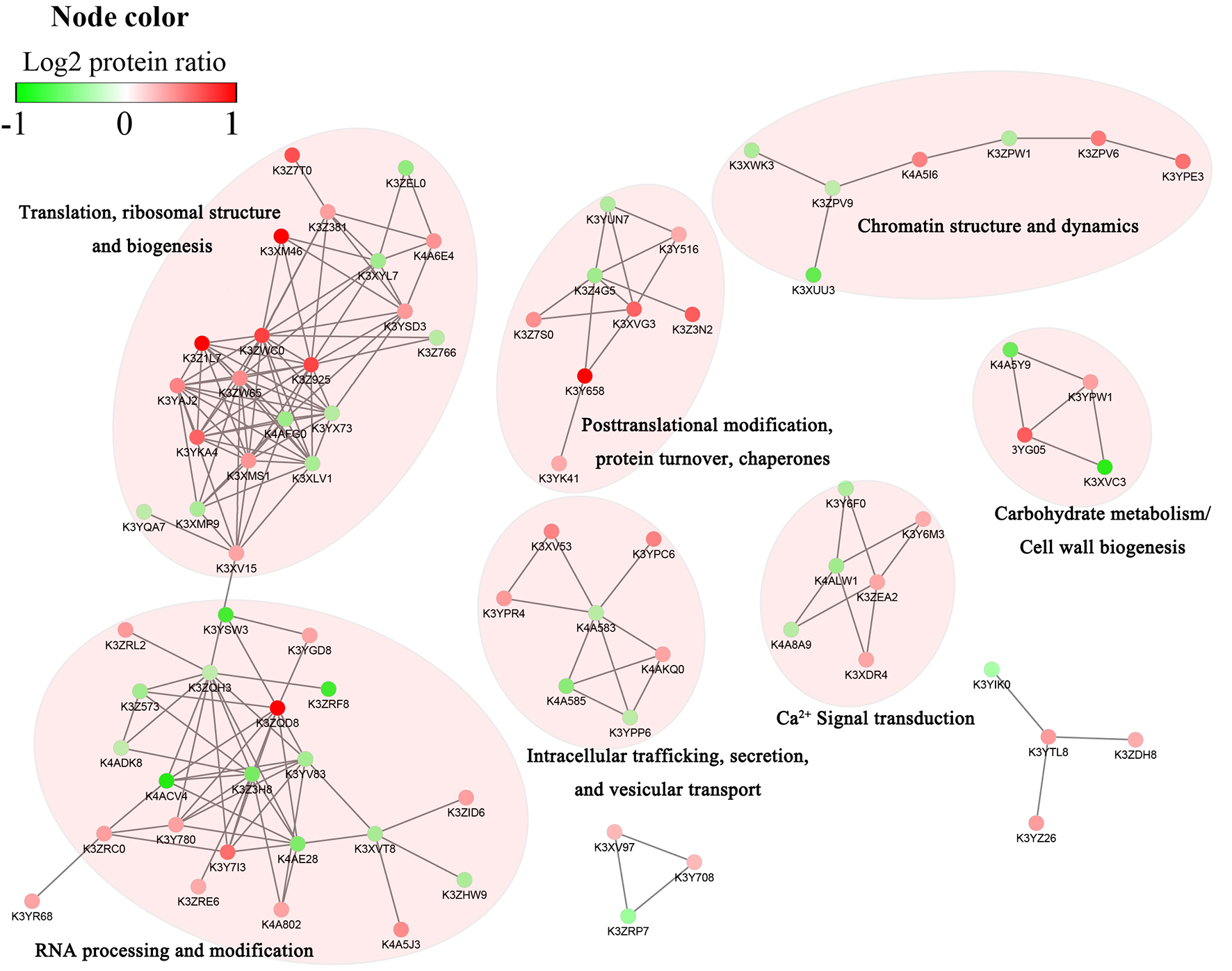

Supplement: Supplementary Figure 7 — Protein–protein interaction networks of DRPPs in six functional categories of An04. [file Image_7.JPEG]
